# Lattice‐Interface Dual Engineering Unlocking Quasi‐Zero‐Strain and High‐Rate Zinc‐Ion Storage in Polyanionic Cathode

**DOI:** 10.1002/advs.202521583

**Published:** 2026-01-20

**Authors:** Qiaofeng Huang, Sheng Ouyang, Jiarui Lin, Rui Jiang, Jiajie Zhou, Xiaoyan Shi, Junling Xu, Lianyi Shao, Zhipeng Sun

**Affiliations:** ^1^ School of Materials and Energy Guangdong University of Technology Guangzhou Guangdong China

**Keywords:** aqueous zinc‐ion batteries, Li doping, Na_3_V_2_O_2_(PO_4_)_2_F, zinc storage mechanism

## Abstract

The advancement of aqueous zinc‐ion batteries is hindered by the performance of cathode. Na_3_V_2_O_2_(PO_4_)_2_F has attracted increasing attention for its advantages, including a high voltage plateau, large ion diffusion channels, and outstanding structural stability. However, its inadequate electronic conductivity causes undesired cycling performance and rate capability. In this work, a microwave hydrothermal‐assisted high‐temperature calcination has been utilized to obtain Li‐doped Na_3_V_2_O_2_(PO_4_)_2_F coated with N‐doped carbon (N_2.85_L_0.15_VOPF@NC). Theoretical calculations and experimental data demonstrate that the co‐modification of Li doping and carbon coating results in favorable morphological integrity, increased electronic conductivity, reduced Zn^2+^ migration barrier, and high average Zn^2+^ diffusion coefficient, contributing to superior electrochemical properties. N_2.85_L_0.15_VOPF@NC exhibits a significantly enhanced performance with reversible capacities of 151.9 and 47.2 mAh g^−1^ at 0.5 and 5 A g^−1^ over 80 and 4,000 cycles, respectively. The soft package batteries also exhibit a stable reversible capacity of 56.4 mAh g^−1^ after 700 cycles. In situ electrochemical impedance spectroscopy uncovers the lattice strain release as an intrinsic factor in capacity enhancement, facilitating the ionic and electrical diffusion processes. In situ X‐ray diffraction, ex situ X‐ray photoelectron spectroscopy, and transmission electron microscopy account for the quasi‐zero‐strain behavior (volume change rate of 1.04%) and the reversible Zn^2+^ insertion/extraction mechanism.

## Graphical abstract

Li‐doped Na_3_V_2_O_2_(PO_4_)_2_F nanoparticles coated with N‐doped carbon are designed to improve their electronic conductivity. The co‐modification of Li doping and carbon coating improves structural integrity, increases electronic conductivity, lowers the Zn^2^^+^ migration barrier, and elevates the average Zn^2^^+^ diffusion coefficient, thereby leading to superior electrochemical performance.

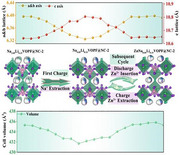

## 1 Introduction

Based on the increasing demand for energy development, the challenges in lithium‐ion batteries have become gradually apparent, such as flammable organic electrolytes and insufficient lithium resources [1, 2, 3, 4, 5, 6]. A notable alternative, rechargeable aqueous zinc ion batteries (AZIBs) have attracted widespread attention for their advantages of low redox potential (−0.76 vs. standard hydrogen electrode), high capacity (820 mAh g^−1^ or 5851 mAh cm^−3^), environmental friendliness, and outstanding safety [7, 8, 9, 10, 11, 12, 13, 14]. Unsatisfactorily, the charge characteristics of the divalent Zn^2+^ lead to heightened electrostatic interactions with the host material, resulting in sluggish reaction kinetics and poor electrochemical performance [15, 16, 17, 18, 19].

At present, cathode candidates for AZIBs include Prussian blue analogs, manganese‐based materials, vanadium‐based materials, organic materials, and polyanionic compounds [20, 21, 22, 23, 24, 25, 26]. Among them, Na_3_V_2_O_2_(PO_4_)_2_F, one of polyanionic compounds, consists of [PO_4_] tetrahedra and [VO_5_F] octahedra forming an open framework structure, which facilitates Zn^2+^ insertion/extraction [27]. The robust phosphate‐oxygen framework grants Na_3_V_2_O_2_(PO_4_)_2_F exceptional structural stability and long lifespan [28, 29]. Furthermore, Na_3_V_2_O_2_(PO_4_)_2_F exhibits a higher charge/discharge plateau than Na_3_V_2_(PO_4_)_3_, attributable to the strong inductive effect of F^−^, enabling improved energy density [30, 31]. However, the primary drawback of these materials is their subpar rate capability due to low intrinsic electronic conductivity (∼ 10^−7^ S cm^−1^), which is ascribed to the V*t_2g_* non‐bonded orbitals causing a wide bandgap of about 2 eV [31, 32].

Typically, carbon coating strategies can significantly improve the surface electronic conductivity. For example, Na_3_V_2_O_2_(PO_4_)_2_F coated by reduced graphene oxide (rGO) delivered a high reversible capacity of 63.9 mAh g^−1^ at 30 C after 5,000 cycles [33]. Besides, element doping is an effective tactic for increasing the intrinsic electronic conductivity. Extensive researches have been devoted to substitutions at the V site in Na_3_V_2‐x_M_x_O_2_(PO_4_)_2_F (M = Mn^3+^, Al^3+^, Mg^2+^, etc.) compounds, achieving a significant improvement in electrochemical properties [18, 34, 35, 36]. Furthermore, replacing inactive Na(1) sites in polyanionic materials with alkali metal ions can also improve intrinsic electronic conductivity and maintain structural stability [37]. Tian et al. successfully prepared K/Co‐doped Na_3_V_2_(PO_4_)_3_ by a sol‐gel method, exhibiting a capacity retention of 75.9% after 400 cycles at 10C [37]. Thus, co‐modification through element doping and surface coating offers the prospect of dual optimization of intrinsic and surface electronic conductivity, enhancing electrochemical performance [35, 38, 39].

Hence, we have employed a microwave hydrothermal method followed by high‐temperature calcination to obtain Li‐doped Na_3_V_2_O_2_(PO_4_)_2_F nanoparticles coated with N‐doped carbon as high‐performance cathode materials for AZIBs. Excellent cycling stability can be achieved with a capacity of 151.9 mAh g^−1^ after 80 cycles at 0.5 A g^−1^ and a retained capacity of 47.2 mAh g^−1^ after 4,000 cycles at 5 A g^−1^. The soft package batteries demonstrate favorable long‐term cycling stability at a high current density of 1 A g^−1^ with a reversible capacity of 56.4 mAh g^−1^ after 700 cycles. Various techniques, including galvanostatic intermittent titration technique (GITT), in situ electrochemical impedance spectroscopy (EIS), and ex situ scanning electron microscopy (SEM), are used to elucidate the reasons behind excellent electrochemical properties. In addition, density functional theory (DFT) calculation indicates that Li doping alters the electronic states near the Fermi level and decreases the Zn^2+^ migration energy barrier. In situ X‐ray diffraction (XRD) evidences excellent structural reversibility and low strain characteristics with an ultra‐small volume change rate of 1.04%. Ex situ X‐ray photoelectron spectroscopy (XPS) and transmission electron microscopy (TEM) further interpret the reversible Zn^2+^ insertion/extraction mechanism.

## 2 Experimental Section

### 2.1 Synthesis of Na_3‐x_Li_x_V_2_O_2_(PO_4_)_2_F Precursor

1.169−x (x = 0, 0.1, 0.15, and 0.20) mmol sodium fluoride (NaF, Aladdin, 98%), x mmol lithium fluoride (LiF, Aladdin, 98%), and 1.05 mmol dihydrogen phosphate (NH_4_H_2_PO_4_, Aladdin, 99%) were dissolved in 12 mL deionized water. 0.7 mmol vanadium acetylacetonate oxide (VO(acac)_2_, Aladdin, 98%) was dissolved in 24 mL ethanol. Subsequently, the above solutions were mixed, stirred, and then reacted in microwave hydrothermal reactor at 130°C for 1 h. The precipitate was washed with ethanol until the solution was clarified. The obtained sediment was dried at 60°C for 12 h.

### 2.2 Synthesis of Na_2.85_Li_0.15_V_2_O_2_(PO_4_)_2_F@NC

Dopamine hydrochloride (Macklin, 98%), polyether (Macklin, 98%), and tris (hydroxymethyl) methyl aminomethane (Accela, ≥ 99%) with a mass ratio of 5:2:5 were dissolved in 100 mL of water together with N_2.85_L_0.15_VOPF (100 mg), and stirred for 24 h. The mixture was filtered, washed with water, and then dried at 60°C for 12 h. Finally, all precursors were placed in a tube furnace filled with nitrogen and heated at 600°C for 7 h. The above samples were labeled as N_3_VOPF, N_2.9_L_0.1_VOPF, N_2.85_L_0.15_VOPF, and N_2.8_L_0.2_VOPF according to different amounts of Li doping. Meanwhile, the samples were labeled as N_2.85_L_0.15_VOPF@NC‐1/2/3, corresponding to dopamine hydrochloride masses of 25, 40, and 70 mg, respectively.

The materials characterization, electrochemical characterization, and computational details are described in the Supporting Information.

## 3 Results and Discussion

**FIGURE 1:**
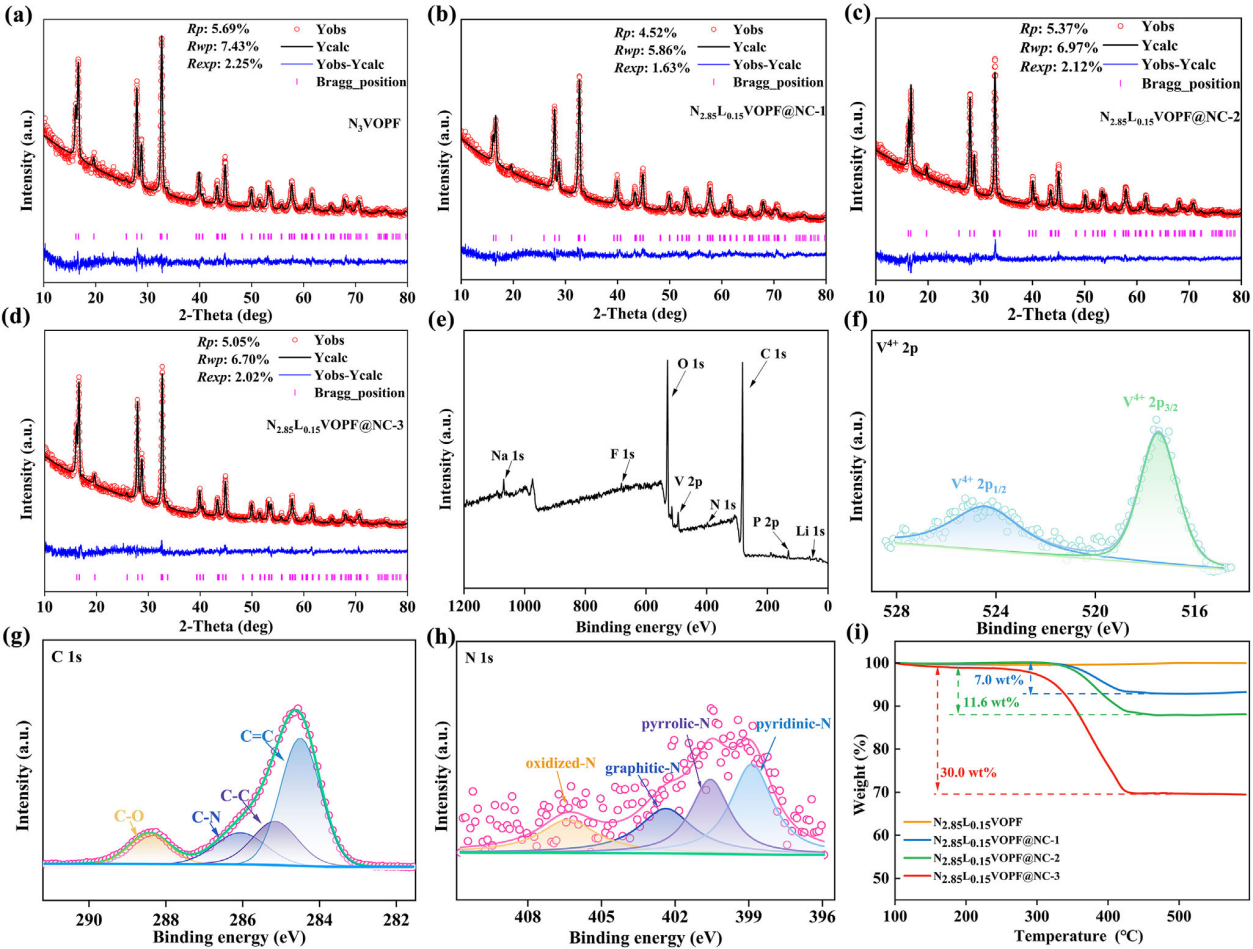
(a–d) Rietveld's refinements of N_3_VOPF and N_2.85_L_0.15_VOPF@NC‐1/2/3. XPS spectra of (e) full spectrum, (f) V 2p, (g) C 1s, and (h) N 1s for N_2.85_L_0.15_VOPF@NC‐2. (i) TG curves.

N3VOPF precursors with different lithium doping amounts were obtained via a microwave hydrothermal method at 130°C for 1 h. Subsequently, N_2.85_L_0.15_VOPF@NC was synthesized by annealing polydopamine‐coated N_2.85_L_0.15_VOPF precursors with different polydopamine contents at 600°C for 7 h under a N_2_ atmosphere. XRD Rietveld's refinement patterns in Figure 1a–d and Figure S1 display reliable fitting factors (*R_p_*, *R_wp_*, and *R_exp_*) for the N_3−x_L_x_VOPF (x = 0.00, 0.10, 0.15, and 0.20) samples, indicating that the introduction of Li^+^ cannot cause the formation of secondary phase. The Li^+^ successfully occupies the Na(1) site, acting as a pillar ion to support the framework [37]. XRD refinement data, including cell parameters and atomic occupancies, are listed in Tables S1–S4. As the amount of the introduced Li^+^ increases, the cell volume slightly decreases from 434.358 (x = 0.00) to 432.448 Å^3^ (x = 0.20) due to the smaller radius of Li^+^ (0.76 Å) compared to Na^+^ (1.02 Å) [40]. Unexpectedly, a single phase and satisfactory fitting factor are also demonstrated in N_2.85_L_0.15_VOPF@NC‐1/2/3.

In Figure 1e, the signals of Na, Li, V, P, O, F, C, and N can be observed in XPS survey spectrum of N_2.85_L_0.15_VOPF@NC‐2, meaning that the polydopamine decomposes into N‐doped carbon during calcination [41, 42]. The proportion of the fitting areas for S_Li_ (152.3)/S_Na_ (2584.4) is approximately 0.058 in Figure S2, supporting the atomic occupancy ratio of Li/Na (0.05) as detailed in Table S3. Inductively coupled plasma‐mass spectrometry (ICP‐MS) data reveals that the measured Li/V molar ratio of 0.104 is close to the designed stoichiometric ratio of 0.075 as shown in Table S5, further indicating the successful synthesis of the target compound. The V 2p spectrum (Figure 1f) displays two distinct peaks at 524.6 and 517.5 eV for V 2p_1/2_ and V 2p_3/2_, respectively, suggesting the presence of V^4+^ [33]. Four matched peaks at 284.5, 285.2, 286.1, and 288.4 eV belong to C═C, C─C, C─N, and C─O in the C 1s spectrum (Figure 1g), respectively [41, 42]. The oxidized‐N, graphitic‐N, pyrrolic‐N, and pyridinic‐N account for the four peaks at 406.4, 402.4, 400.5, and 398.9 eV in the high‐resolution N 1s spectrum (Figure 1h), respectively [42, 43]. These results confirm the successful nitrogen doping into the carbon layer via polydopamine transformation after high‐temperature treatment. Besides, the N_2.85_L_0.15_VOPF@NC‐1/2/3 samples contain carbon contents of 7.0, 11.6, and 30.0 wt.% according to the TG curves in Figure 1i. The partial replacement of Li^+^ can enhance structural stability, while appropriate surface coating can collectively improve the electrochemical properties [37].

**FIGURE 2:**
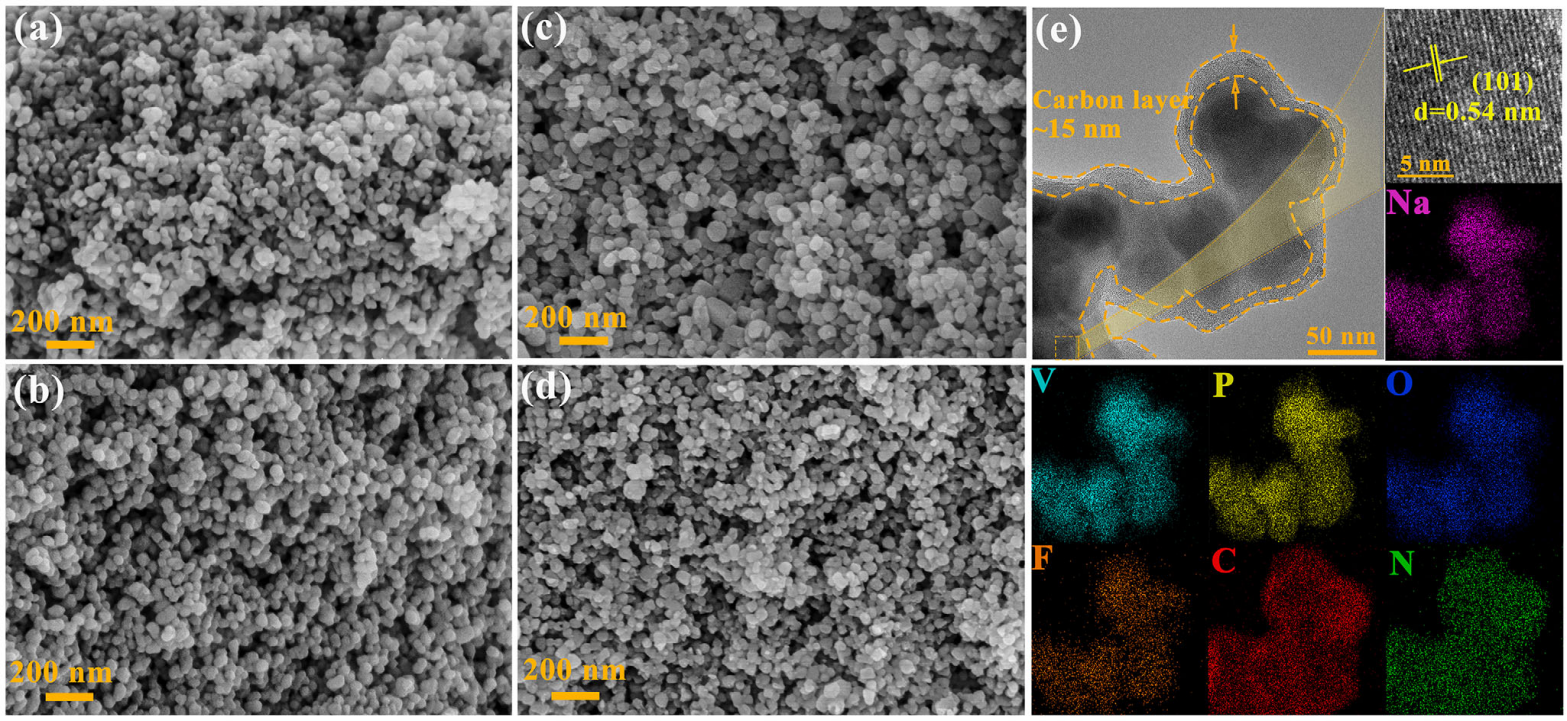
SEM images of (a) N_3_VOPF and (b–d) N_2.85_L_0.15_VOPF@NC‐1/2/3. (e) TEM, HRTEM, and EDS mapping images for N_2.85_L_0.15_VOPF@NC‐2.

SEM and TEM techniques were employed to analyze the microstructure and morphology of N_3‐x_L_x_VOPF and N_2.85_L_0.15_VOPF@NC‐1/2/3. Figure 2a, Figures S3, and S4 reveal that the introduction of Li^+^ shows a negligible impact on particle morphology and size, which falls within the range of 30–50 nm. Upon N‐doped carbon coating, the particle size increases to approximately 40–70 nm as the N_2.85_L_0.15_VOPF particles are enveloped by a thin N‐doped carbon layer with the thickness of about 15 nm (Figure 2b–e; Figure S5). The (101) lattice plane with a lattice spacing of 0.54 nm (JCPDS 04‐011‐1042) is also observed. Additionally, the energy dispersive spectroscopy (EDS) mapping images confirm the uniform distribution of Na, V, P, O, F, C, and N throughout the N_2.85_L_0.15_VOPF@NC‐2 composite. The N‐doped carbon coated on the surface of the particles not only increases surface electronic conductivity but also inhibits vanadium dissolution by preventing direct contact between electrolyte and active particles [44].

To estimate the zinc ion storage performance of the synthesized cathodes, a 2 M Zn(OTf)_2_ + 4 M LiOTf solution was employed as the aqueous electrolyte. Our previous work suggested that LiOTf, as an electrolyte additive, can improve the oxygen evolution reaction, increase ionic conductivity, and inhibit vanadium dissolution [33]. Galvanostatic charge–discharge (GCD) curves of N_3‐x_L_x_VOPF in Figure S6 demonstrate two charge plateaus at around 1.63 and 1.80 V, corresponding to the release of two Na(2) ions from the host material. Subsequent Zn^2+^ insertion occurs through a single‐step process, resulting in a discharge slope with an average potential of 1.52 V. Notably, only one charge/discharge slope is evident in the 2^nd^ and 3^rd^ cycles, further demonstrating the extraction/insertion of Zn^2+^, which differs from the two‐plateau mechanism observed in sodium ion batteries [28, 30]. N_2.85_L_0.15_VOPF sample maintains a capacity of 109.9 mAh g^−1^ after 40 cycles at 0.1 A g^−1^ in Figure S7, significantly outperforming the values for N_3_VOPF (71.5 mAh g^−1^), N_2.9_L_0.1_VOPF (96.6 mAh g^−1^), and N_2.8_L_0.2_VOPF (56.8 mAh g^−1^). A remarkable enhancement in rate capability is observed for N_2.85_L_0.15_VOPF sample, delivering capacities of 117.4, 96.5, 83.7, 74.4, 61.1, and 47.8 mAh g^−1^ at 0.1, 0.2, 0.5, 1, 2, and 4 A g^−1^, respectively. Even at an increased current density of 5 A g^−1^ (∼38C), a capacity of 43.1 mAh g^−1^ is achieved, recovering to 100.8 mAh g^−1^ when the current density is returned to 0.1 A g^−1^. In contrast, N_3‐x_L_x_VOPF samples with x = 0.00, 0.10, and 0.20 exhibit poor capacities of just 20.8, 19.4, and 15.3 mAh g^−1^ at 5 A g^−1^.

**FIGURE 3:**
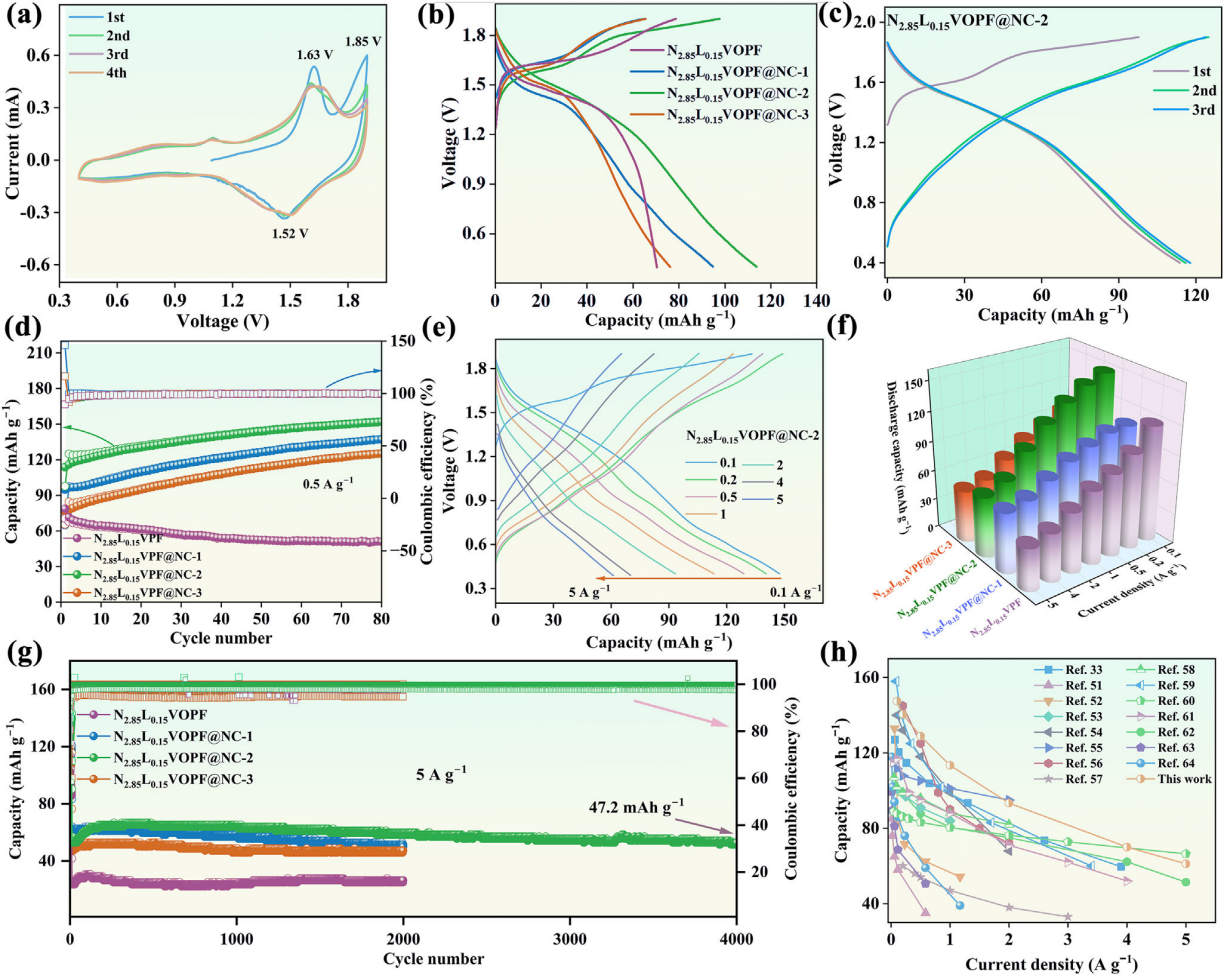
(a) CV curves of N_2.85_L_0.15_VOPF@NC‐2 at 0.2 mV s^−1^. (b) Initial GCD profiles of N_2.85_L_0.15_VOPF and N_2.85_L_0.15_VOPF@NC‐1/2/3 at 0.5 A g^−1^. (c) GCD curves of N_2.85_L_0.15_VOPF@NC‐2 for the first three cycles at 0.5 A g^−1^. (d) Cycling performance at 0.5 A g^−1^. (e) GCD curves of N_2.85_L_0.15_VOPF@NC‐2 at different current densities from 0.1 to 5 A g^−1^. (f) Rate performance. (g) Cycling performance at 5 A g^−1^. (h) Comparison of rate capability between N_2.85_L_0.15_VOPF@NC‐2 and the reported materials.

The improvement in electrochemical performance attributed to Li doping is still limited. The incorporation of N‐doped carbon on N_2.85_L_0.15_VOPF can further improve its cycling stability and rate performance. In the initial CV and dQ/dV curves (Figure 3a; Figure S8), the anodic peaks at 1.63 and 1.85 V and the cathodic peak at 1.52 V can correspond to the extraction of Na^+^ and the insertion of Zn^2+^, respectively, consistent with the GCD results [15, 45]. The curves exhibits good consistency and excellent overlap after the first cycle, along with a distinct redox couple at 1.63/1.52 V, indicating high electrochemical reversibility. N_2.85_L_0.15_VOPF@NC‐2 exhibits the highest initial discharge capacity of 113.8 mAh g^−1^ at 0.5 A g^−1^, shown in Figure 3b, while N_2.85_L_0.15_VOPF and N_2.85_L_0.15_VOPF@NC‐1/3 exhibit lower capacities of 78.6, 94.8, and 76.2 mAh g^−1^, respectively. An enhancement in Coulombic efficiency along with the repeated cycles is observed in Figure 3c and Figure S9. For instance, the Coulombic efficiency of N_2.85_L_0.15_VOPF@NC‐2 increases from 92.9% at the 2^nd^ cycle to 95.1% at the 3^rd^ cycle. Obviously, the capacity of N_2.85_L_0.15_VOPF@NC‐2 reaches 151.9 mAh g^−1^ after 80 cycles (Figure 3d) [46, 47]. The capacities of N_2.85_L_0.15_VOPF@NC‐1/3 are 137.0 and 125.1 mAh g^−1^ under the same conditions, whereas the capacity of N_2.85_L_0.15_VOPF significantly decreases to 51.0 mAh g^−1^. The carbon coating contributes to enhanced capacity, in some cases exceeding the theoretical value. This improvement is primarily attributed to the N‐doped carbon layer, which not only provides additional pseudocapacitive charge storage at the electrode–electrolyte interface, but also improves ionic accessibility and interfacial kinetics, thereby facilitating the progressive activation of deeper redox‐active sites [29, 48, 49]. Furthermore, the gradual infiltration of the electrolyte into the electrodes during cycling is another reason for activation [46, 47, 50]. Figure 3e,f, and Figure S10 also demonstrate a substantial enhancement in rate performance for N_2.85_L_0.15_VOPF@NC‐2 relative to N_2.85_L_0.15_VOPF, which can remain stable capacities of 147.4, 140.4, 128.9, 113.4, 93.4, 70.0, and 61.1 mAh g^−1^ at current densities ranging from 0.1 to 5 A g^−1^, respectively. Moreover, N_2.85_L_0.15_VOPF@NC‐2 retains a capacity of 47.2 mAh g^−1^ after 4,000 cycles at a high current density of 5 A g^−1^, whereas N_2.85_L_0.15_VOPF only delivers a capacity of 26.4 mAh g^−1^ after 2,000 cycles, as shown in Figure 3g. The above electrochemical characteristics imply that the co‐modification effect of N‐doped carbon coating and Li^+^ doping effectively preserves structural stability and enhances electrochemical reversibility of the cathode material. Hence, N_2.85_L_0.15_VOPF@NC‐2 conveys notable improvement in rate performance compared to previously reported vanadium phosphate materials (Figure 3h) [33, 51, 52, 53, 54, 55, 56, 57, 58, 59, 60, 61, 62, 63, 64].

**FIGURE 4:**
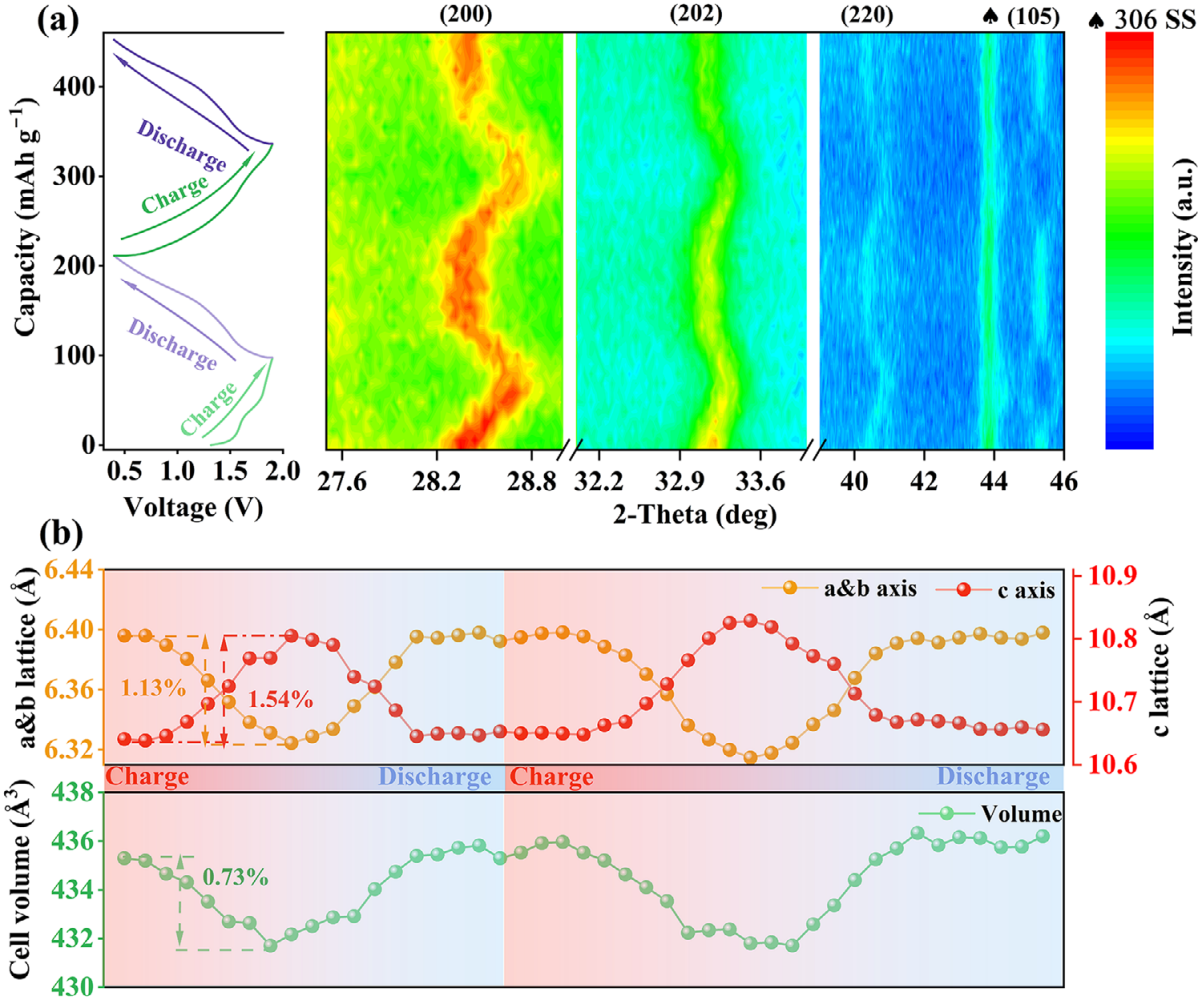
(a) In situ XRD patterns of N_2.85_L_0.15_VOPF@NC‐2 for the initial two cycles. (b) Cell parameters and volume variation calculated by XRD Rietveld's refinement.

The intriguing electrochemical zinc storage mechanism was explored through in situ XRD to monitor structural evolution during cycling, while ex situ XPS and TEM were applied to observe the changes in atomic information at different states of charge (SOC). Ex situ SEM was performed to analyze morphological variations at different cycles. In situ XRD patterns and GCD curves are presented in Figure 4a and Figure S11, revealing reversible shifts in the (200), (202), (220), and (105) crystal planes due to Na^+^ extraction (1^st^ charging) and subsequent Zn^2+^ extraction/insertion. The corresponding cell parameters at different SOC are obtained through XRD Rietveld's refinement as shown in Figure 4b. Throughout the initial charging, the a‐ and b‐axis slowly contract from 6.396 to 6.324 Å with a variation rate of 1.13%, while the c‐axis indicates a slight increase from 10.641 to 10.805 Å with a variation rate of 1.54%, leading to a reduction in cell volume from 435.311 to 432.124 Å^3^ (correlative change rate of 0.73%), attributed to Na^+^ extraction‐induced changes. The subsequent discharging process restores the original framework, with axis returning to 6.398 Å (a,b) and 10.647 Å (c), and volume recovering to 435.829 Å^3^. Remarkably, the second cycle reveals nearly identical charged‐state volume (431.700 Å^3^) and minimal volume fluctuation (1.04%), demonstrating exceptional structural reversibility. The cumulative volume expansion to 436.200 Å^3^ after two cycles suggests internal stress relaxation and lattice activation, correlating with reduced charge transfer impedance (R_ct_) value and stabilized Zn^2+^ diffusion coefficient (*D_Zn_^2+^*) as discussed later. These results collectively confirm the quasi‐zero strain characteristics for this material during cycling.

**FIGURE 5:**
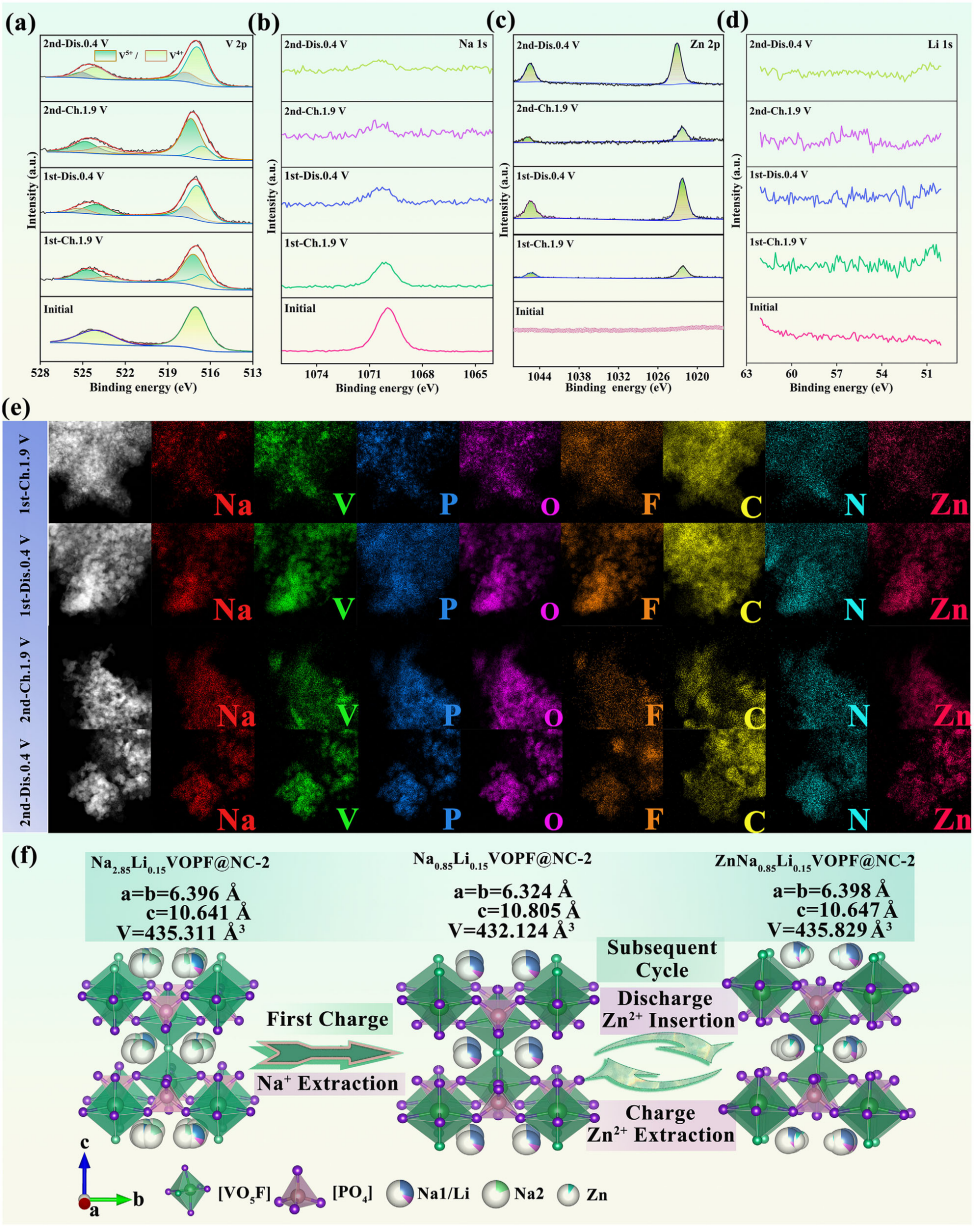
Ex situ XPS of (a) V 2p, (b) Na 1s, (c) Zn 2p, and (d) Li 1s. (e) Ex situ TEM EDS mapping images at different states. (f) Schematic illustrations of ion insertion/extraction in N_2.85_L_0.15_VOPF@NC‐2.

Ex situ XPS was employed to characterize the alterations in the V, Na, Zn, and Li elements of N_2.85_L_0.15_VOPF@NC‐2 cathode materials at different SOC. Upon initial charging to 100% SOC, the valence state of V is converted from +4 to +5 due to the release of Na^+^ in Figure 5a,b. The weak signal in the Na 1s region originates from residual Na^+^ ions occupying electrochemically inactive Na(1) sites, as evidenced by minimal signal changes during subsequent cycling. Discharging to 0.4 V, an apparent increase in the Zn 2p signal is observed in Figure 5c, indicating the conversion of V^5+^ to V^4+^ while Zn^2+^ ions enter into the Na sites. This reversible redox behavior persists throughout the 2^nd^ cycle. Moreover, the absence of Li 1s signal on the electrode surface demonstrates that the Li^+^ is still free in the electrolyte, consistent with previous reports [33]. Ex situ TEM further corroborates Zn^2+^ participation in the electrochemical process. The minimal presence of Zn^2+^ at the 100% SOC in the initial cycle stems from residual electrolyte on the electrode surface (Figure 5e), corresponding to the XPS results observed in Figure 5c. When discharged to 0.4 V, the pronounced Zn signals imply substantial Zn^2+^ insertion. The residual Zn^2+^ in the subsequent charged state can act as a support for stabilizing the 3D framework of the material [65]. The absence of the characteristic peaks for Zn_x_(CF_3_SO_3_)_y_(OH)_2x‐y_⋅nH_2_O (12.9°, 19.4°, 25.9°, and 32.4°) observed from in situ XRD and no observable flaky by‐product in SEM images (Figure S12) collectively confirm the proton‐free nature of the electrochemical reaction [66, 67, 68]. Ex situ SEM images also reveal excellent morphological retention of particles through 50 cycles, facilitating cycling stability. Hence, the Zn^2+^ insertion/extraction mechanism is summarized in Figure 5f.

**FIGURE 6:**
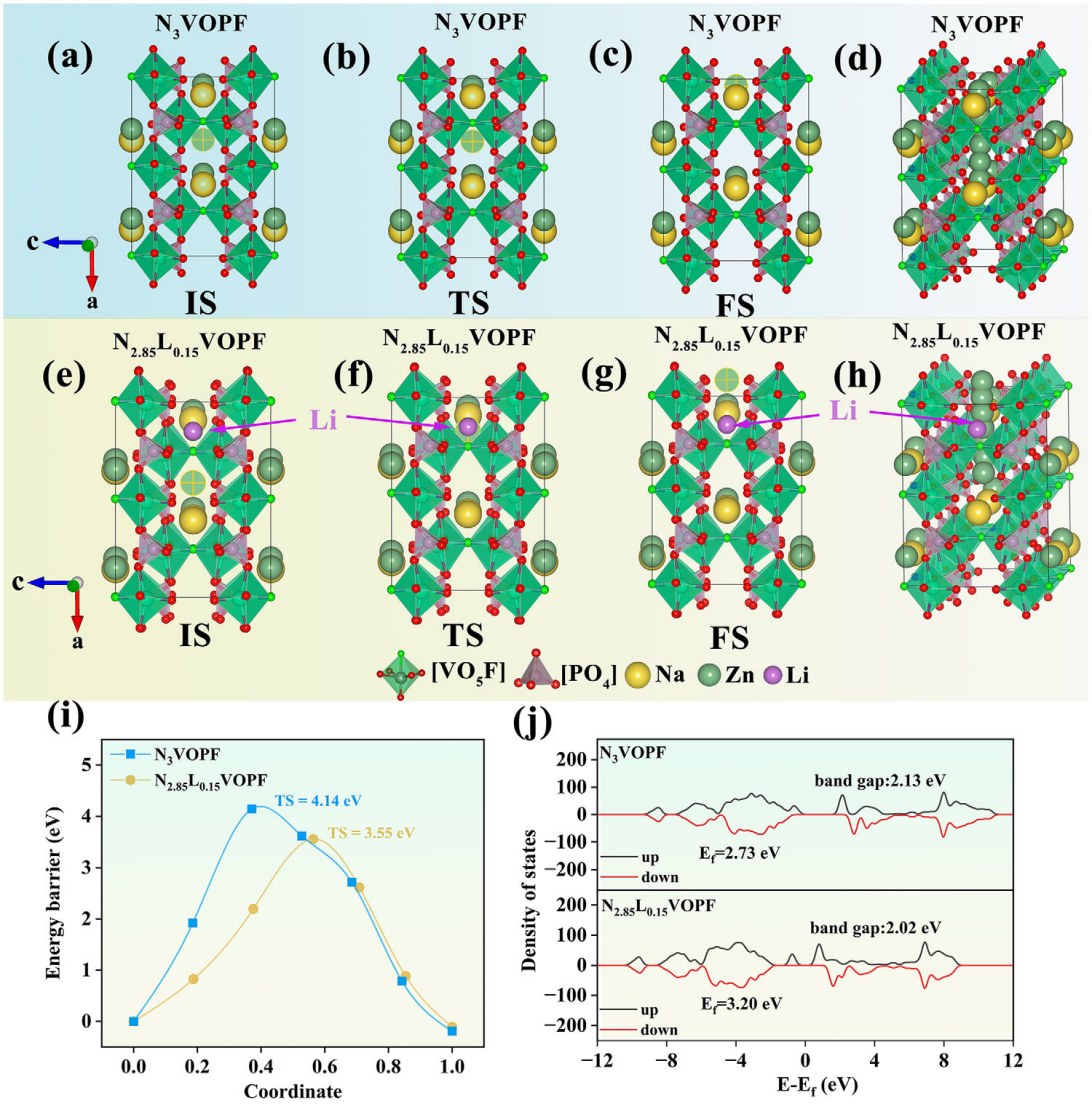
Zn^2+^ migration pathways in N_3_VOPF (a–d) and N_2.85_L_0.15_VOPF (e–h). (i) The corresponding migration energy profiles for N_3_VOPF and N_2.85_L_0.15_VOPF. (j) Density of states for N_3_VOPF and N_2.85_L_0.15_VOPF.

To further understand the relationship of Li doping on the crystal structure and electrochemical properties of N_3_VOPF, DFT calculations were used to investigate the band gap widths and Zn^2+^ migration energy barriers of N_3_VOPF and N_2.85_L_0.15_VOPF. The optimized crystal structures of N_3_VOPF and N_2.85_L_0.15_VOPF before and after Zn^2+^ substitution at the Na(2) sites, along with the corresponding Zn^2+^ diffusion paths, are obtained in Figures S13 and S14, where the Zn^2+^ diffusion pathways are mainly concentrated in the a and b‐axes. Figure 6 represents the structures with different Zn^2+^ insertion states for N_3_VOPF and N_2.85_L_0.15_VOPF, where IS, TS, and FS stand for the initiation state, transition state, and termination state, respectively. The migration energy barriers for N_3_VOPF and N_2.85_L_0.15_VOPF are calculated to be 4.14 and 3.55 eV, respectively, indicating a faster Zn^2+^ diffusion for N_2.85_L_0.15_VOPF. To elucidate the enhanced performance after Li doping, the band structure and density of states of N_3_VOPF and N_2.85_L_0.15_VOPF were further simulated using the DFT approach in the Vienna Ab initio Simulation Package (VASP). As illustrated in Figure 6j, N_2.85_L_0.15_VOPF manifests a bandgap width of 2.02 eV, which is lower than the value for N_3_VOPF (2.13 eV). Moreover, the Fermi level of N_2.85_L_0.15_VOPF (3.20 eV) is higher than that of N_3_VOPF (2.73 eV), signifying the effective enhancement of intrinsic electronic conductivity after Li doping. In conclusion, the theoretical calculations suggest that the superior performance of N_2.85_L_0.15_VOPF is attributed to Li doping, which simultaneously reduces the ionic migration barrier and improves electronic conductivity.

**FIGURE 7:**
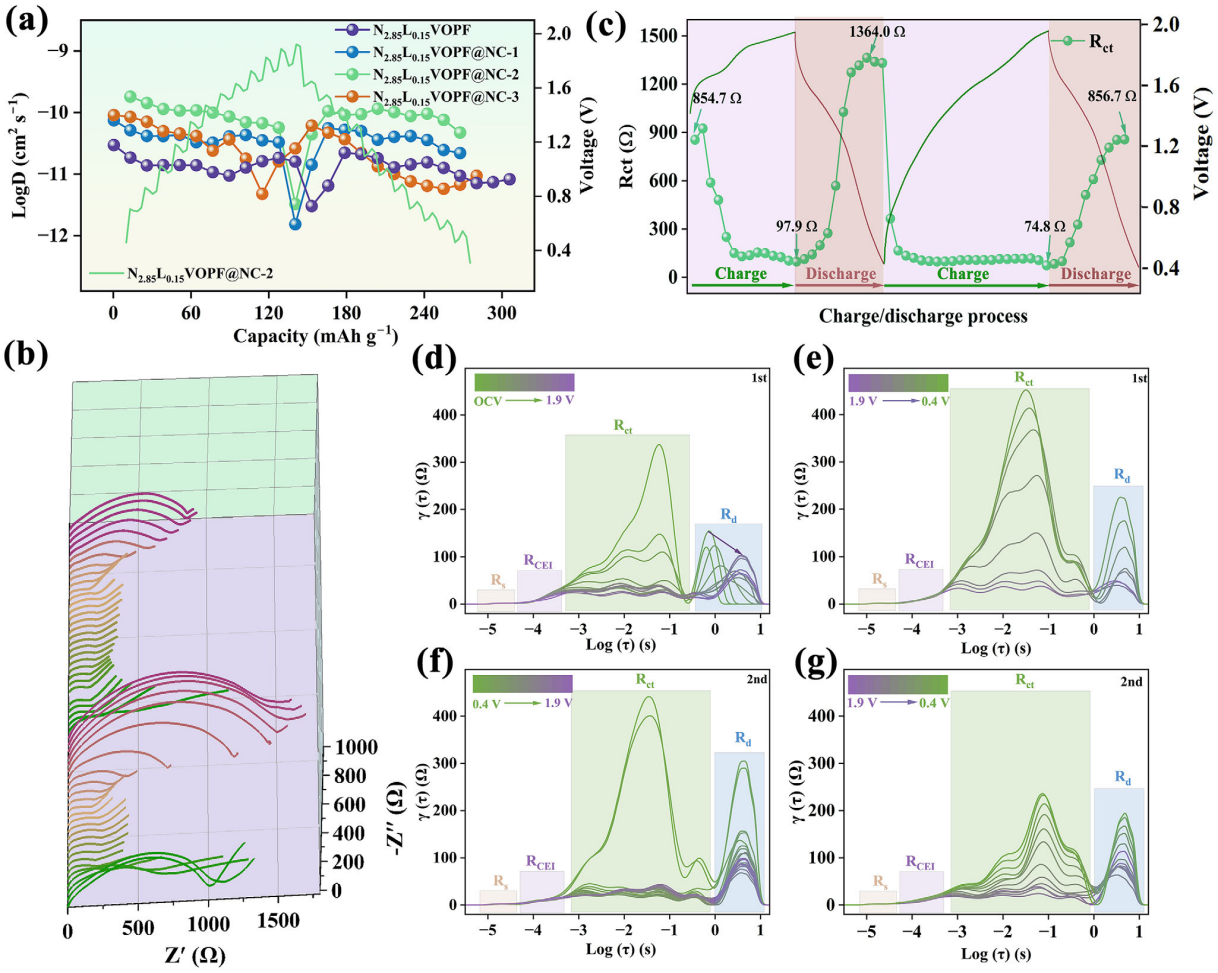
(a) Zn^2+^ diffusion coefficient curves of N_2.85_L_0.15_VOPF and N_2.85_L_0.15_VOPF@NC‐1/2/3. (b) In situ EIS curves of N_2.85_L_0.15_VOPF@NC‐2. (c) In situ EIS fitting results. (d–g) DRT plots calculated from EIS measurements at different potentials.

With the purpose of explaining the excellent electrochemical properties of N_2.85_L_0.15_VOPF@NC‐2, the galvanostatic intermittent titration technique (GITT) and in situ EIS were employed to determine the *D_Zn_^2+^* and R_ct_ values. In Figure 7a, a stable D*_Zn_^2+^* is observed for N_2.85_L_0.15_VOPF@NC‐2 with a calculated average value of 1.89 × 10^−9^ cm^2^ s^−1^, surpassing the data for N_2.85_L_0.15_VOPF (1.38 × 10^−11^ cm^2^ s^−1^), N_2.85_L_0.15_VOPF@NC‐1 (8.44 × 10^−10^ cm^2^ s^−1^), and N_2.85_L_0.15_VOPF@NC‐3 (7.35 × 10^−10^ cm^2^ s^−1^). Besides, N_2.85_L_0.15_VOPF@NC‐2 exhibits an enhanced conductivity of 3.75 × 10^−5^ S cm^−1^, compared to N_2.85_L_0.15_VOPF (2.26 × 10^−5^ S cm^−1^) through four‐point probe measurements, as shown in Table S6, demonstrating the effectiveness of the conductive carbon layer in facilitating electron transport. In situ EIS curves (Figure 7b) in the initial two cycles indicate a gradual decrease in the R_ct_ value during the charging process and a stepwise increase during the discharging process. The detailed R_ct_ variations are obtained through equivalent circuit fitting in Figure 7c, revealing that the R_ct_ value decreases from 854.7 Ω at open‐circuit potential to 97.9 Ω at the charging cut‐off voltage of 1.9 V owing to the facilitated Na^+^ extraction through the open structure. Upon discharging, the value increases to 1364.0 Ω, attributed to the strong electrostatic interactions between Zn^2+^ and host material. When discharged to 0.4 V in the second cycle, the R_ct_ value reduces to 856.7 Ω owing to the release of lattice strain as supported by in situ XRD. DRT analysis (Figure 7d–g) resolves four distinct electrochemical processes, including contact impedance (R_s_, time relaxation constant τ: 10^−5.3^–10^−4.5^ s), ionic diffusion impedance at the cathode‐electrolyte interface (R_CEI_, τ: 10^−4.5^–10^−3.5^ s), charge‐transfer impedance (R_ct_, τ: 10^−3.5^–1 s), and zinc ion diffusion impedance (R_d_, τ: 1–10 s) [63, 69]. Among them, R_s_ and R_CEI_ exhibit low impedance and good stability during cycling, indicating good contact between the interfaces. Notably, a significant increase in R_ct_ and R_d_ during the initial discharging process indicates strong electrostatic interactions of Zn^2+^ with the host material, however, the bottleneck effect of R_ct_ and R_d_ is significantly weakened upon the second discharging, suggesting that the host material is fully stress released and therefore acquires superior electron/ion transport channels to improve the diffusion kinetics. Thus, the stability of *D_Zn_^2+^* ensures superior electrochemical properties, while R_ct_ fluctuations reflect the activation process and potentially serve as an intrinsic factor that influences capacity enhancement by releasing lattice strain to access more suitable ionic and electrical diffusion channels [40, 70, 71].

**FIGURE 8:**
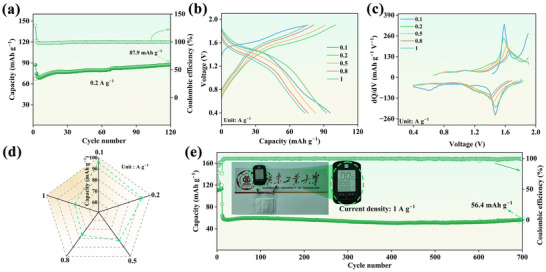
(a) Cycling performance of soft package batteries at 0.2 A g^−1^. (b) GCD curves. (c) The corresponding dQ/dV curves at various current densities. (d) Rate performance. (e) Cycling performance at 1 A g^−1^ (activated at 0.1 A g^−1^ for 10 cycles. Insert: Walker sports recorder activated by two soft package batteries in series).

To demonstrate the practical potential of N_2.85_L_0.15_VOPF@NC‐2 cathode material, soft package cells were explored with the following specifications: an effective area of 3.2 × 3.5 cm^2^, an active material loading mass of 12–15 mg, and an electrolyte injection volume of 1.8 mL. The initial discharge capacity is 87.1 mAh g^−1^ at 0.2 A g^−1^, showing a slight rise to 87.9 mAh g^−1^ after 120 cycles (Figure 8a). The GCD and differential capacitance (dQ/dV) curves exhibit small polarization voltages across various current densities (Figure 8b,c), guaranteeing enhanced energy densities. As illustrated in Figure 8d and Figure S15, the capacity is maintained at 95.7, 92.2, 82.7, 76.2, and 73.3 mAh g^−1^ at current densities of 0.1, 0.2, 0.5, 0.8, and 1 A g^−1^, respectively. The calculated energy and power densities of the soft package cells are compared with other reported cells in the Ragone plot (Figure S16), further highlighting the practical competitiveness of our device [29, 72, 73, 74, 75]. Equally, the excellent cycling performance is achieved at high current density, as depicted in Figure 8e. After activating at 0.1 A g^−1^ over 10 cycles, the cell maintains a capacity of 56.4 mAh g^−1^ after 700 cycles at 1 A g^−1^, demonstrating exceptional cycling stability. Moreover, the walker sports recorder with a voltage rating of 3.4 V can work properly after connecting two soft package batteries. These results highlight the strong potential of this material for energy storage applications owing to its high capacity, elevated voltage, and exceptional cycling durability.

## 4 Conclusion

To summarize, we have successfully prepared N‐doped carbon‐coated and Li‐doped Na_3_V_2_O_2_(PO_4_)_2_F nanoparticles, N_2.85_L_0.15_VOPF@NC‐2, as cathode materials for AZIBs by a microwave hydrothermal‐assisted high‐temperature annealing method. The N_2.85_L_0.15_VOPF@NC‐2 electrode exhibits excellent electrochemical properties, possessing a capacity of 151.9 mAh g^−1^ over 80 cycles at 0.5 A g^−1^ and maintaining a capacity of 47.2 mAh g^−1^ after 4,000 cycles at 5 A g^−1^ (∼38C). The remarkable cycling stability is also demonstrated in soft package battery with a reversible capacity of 56.4 mAh g^−1^ at 1 A g^−1^ after 700 cycles. The favorable morphological integrity, enhanced electronic conductivity, reduced Zn^2+^ migration barrier, and high average *D_Zn_^2+^* collectively contribute to the excellent electrochemical properties. In situ EIS reflects lattice strain release as an intrinsic factor influencing capacity enhancement. Regarding the Zn^2+^ storage mechanism section, in situ XRD exhibits ultra‐high structural stability with quasi‐zero‐strain characteristics (an ultra‐small volume change rate of 1.04%). Ex situ XPS and TEM further illustrate a reversible Zn^2+^ insertion/extraction mechanism. Therefore, N_2.85_L_0.15_VOPF@NC‐2 is a promising high‐rate cathode material for AZIBs.

## Author Contributions

**Qiaofeng Huang**: performed investigation, wrote the original draft, edited the final manuscript, and data curation. **Sheng Ouyang**: performed investigation, wrote the original draft, and edited the final manuscript. **Jiarui Lin**, **Rui Jiang**, **Jiajie Zhou**, **Xiaoyan Shi**, and **Junling Xu**: performed data curation. **Lianyi Shao**: conceived the research, wrote the original draft, edited the final manuscript, supervision, and funding acquisition. **Zhipeng Sun**: performed supervision and funding acquisition.

## Conflicts of Interest

The authors declare no conflict of interest.

## Supporting information




**Supporting File**: advs73876‐sup‐0001‐SuppMat.docx.

## Data Availability

The data that support the findings of this study are available in the supplementary material of this article.
