# Supplementary material for: Lattice‐Interface Dual Engineering Unlocking Quasi‐Zero‐Strain and High‐Rate Zinc‐Ion Storage in Polyanionic Cathode
Source: Adv Sci (Weinh). 2026 Jan 20;13(17):e21583. doi: 10.1002/advs.202521583 (PMC13042900; doi:10.1002/advs.202521583)
Supplement: Supplementary file 1 — Supporting File: advs73876‐sup‐0001‐SuppMat.docx. [file ADVS-13-e21583-s001.docx]

**Lattice-Interface Dual Engineering Unlocking Quasi-Zero-Strain and High-Rate Zinc-Ion Storage in Polyanionic Cathode**

*Qiaofeng Huang,* *Sheng Ouyang, Jiarui Lin, Rui Jiang, Jiajie Zhou, Xiaoyan Shi,* *Junling Xu, Lianyi Shao,^*^ Zhipeng Sun^*^*

School of Materials and Energy, Guangdong University of Technology, Guangzhou 510006, Guangdong, China. E-mail: [shaolianyi@gdut.edu.cn](mailto:shaolianyi@gdut.edu.cn); [zpsunxj@gdut.edu.cn](mailto:zpsunxj@gdut.edu.cn)

**Experimental section**

**Materials characterization**

XRD patterns were collected by X-ray diffractometer with Cu Kα radiation (λ = 1.5418 Å). The morphology and microstructure of the as-synthesized products were characterized through SEM (JEOL-6300F) and TEM (Talos F200S, Thermo). XPS experiment was carried out with an AXIS SUIPRA^+^ spectrometer. Raman spectra were obtained using a micro‐Raman spectrometer (Renishaw inVia Qontor) with a 532 nm laser for excitation. Thermal gravimetric (TG) analysis was performed by a TGA 4000 instrument in air at a heating rate of 10 °C min^−1^. The Li/V ratio was determined by inductively coupled plasma-mass spectrometry (ICP-MS, Thermo Fisher). The electrical conductivities of the samples were measured using a four-point probe system (RTS-9).

**Electrochemical characterization**

The electrode slurry was acquired by mixing the active material, polyvinylidene fluoride (PVDF), and super P in a mass ratio of 7:2:1 with a suitable proportion of N-methyl-2-pyrrolidone (NMP) in a mortar and thoroughly grinding. The cathode electrode was fabricated by coating the slurry onto a 306 stainless steel mesh (12 mm diameter) and drying at 60 °C for 10 h, resulting in a mass loading of approximately 2 mg cm^−2^. A glass fiber was employed as a separator, zinc foil was used as an anode, and 100 uL solution composed of 2 M zinc trifluoromethane sulfonate (Zn(OTf)_2_) and 4 M lithium trifluoromethane sulfonate (LiOTf) was applied as an aqueous electrolyte. The electrochemical properties were measured using the Neware battery testing system (CT-4008T). Cyclic voltammetry (CV, 0.4–1.9 V) and EIS (0.01–10^5^ HZ) were conducted by the Chenhua CHI Electrochemical Workstation (CHI 760E) at room temperature.

**Computational details**

All the calculations in this work were performed using the Vienna Ab initio Simulation Package (VASP).^[1]^ The valence electron and core-ion interactions were described using the projected augmented wave (PAW).^[2]^ The electron exchange and correlation energies were calculated using the generalized gradient approximation/Perdew–Burke–Ernzerhof (GGA/PBE) exchange-correlation functional.^[3]^ A cutoff energy of 450 eV was applied to the plane wave basis expansion. To describe the strong electron-correlation effect on the V 3d orbitals, the Hubbard GGA+U model was used with an effective *U*_eff_ value of 3.25 eV. A 2 × 2 × 3 Monkhorst-Pack k-point grid was used for the integration over the first Brillouin zone. The lattice parameters and ionic positions were fully relaxed until the total energies and ionic forces were less than 10^–5^ eV and 0.02 eV Å^–1^, respectively. The Zn^2+^ self-diffusion was calculated using the climbing image nudged elastic band (CI-NEB) method to seek for the saddle points and minimum energy paths.^[4]^


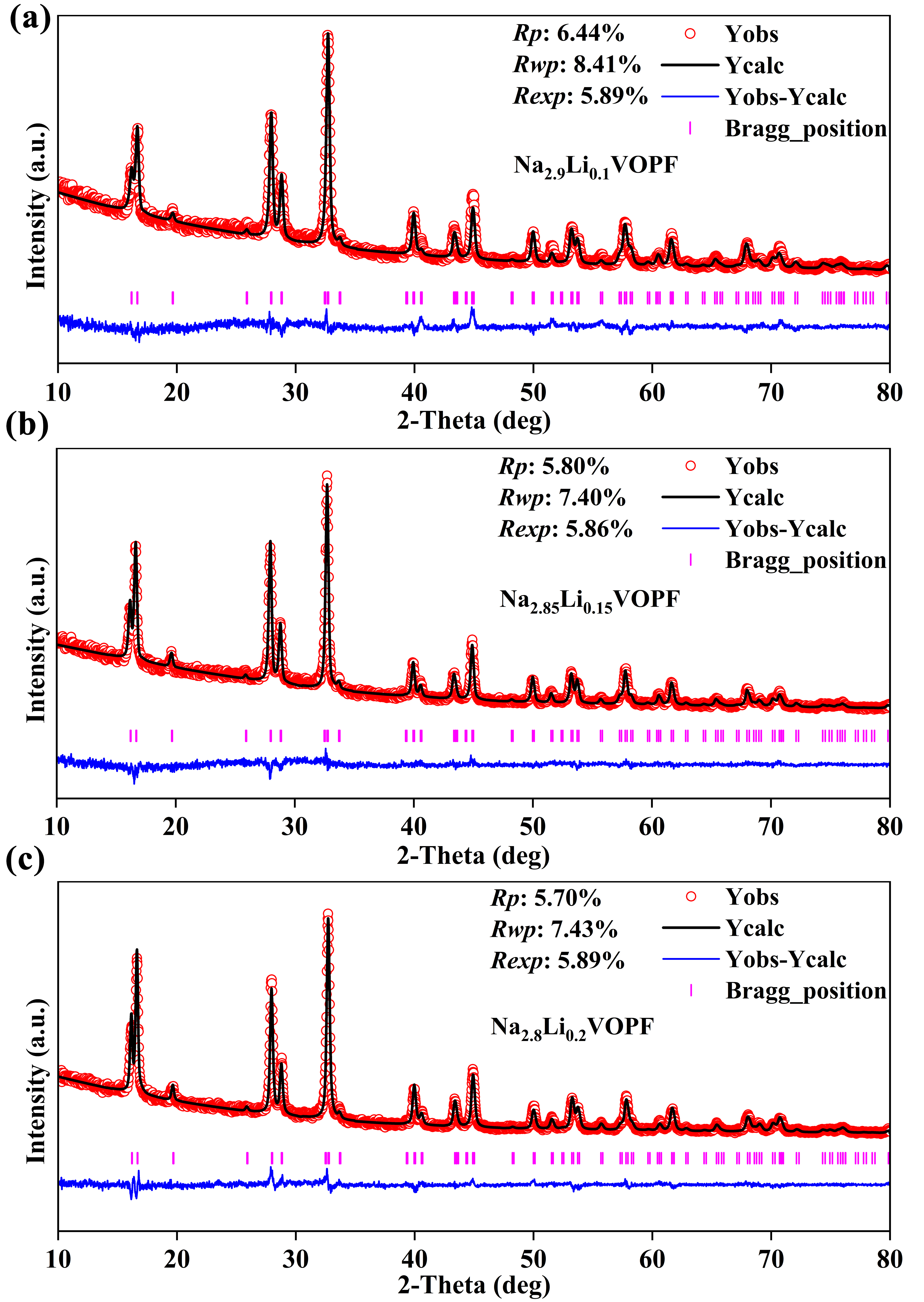


**Figure S1.** Rietveld’s refinement patterns of (a) Na_2.9_Li_0.1_VOPF, (b) Na_2.85_Li_0.15_VOPF, and (c) Na_2.8_Li_0.2_VOPF.

**
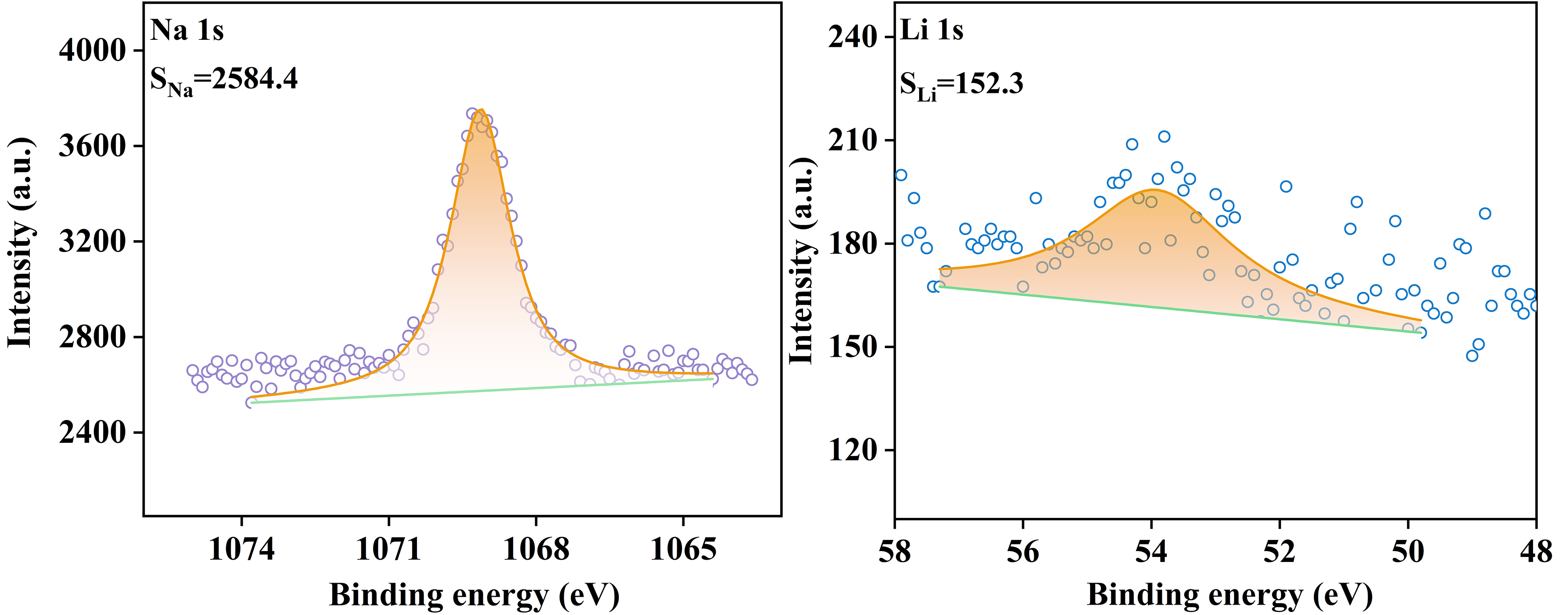
Figure S2.** XPS curves of (a) Na 1s and (b) Li 1s for N_2.85_L_0.15_VOPF@NC-2.


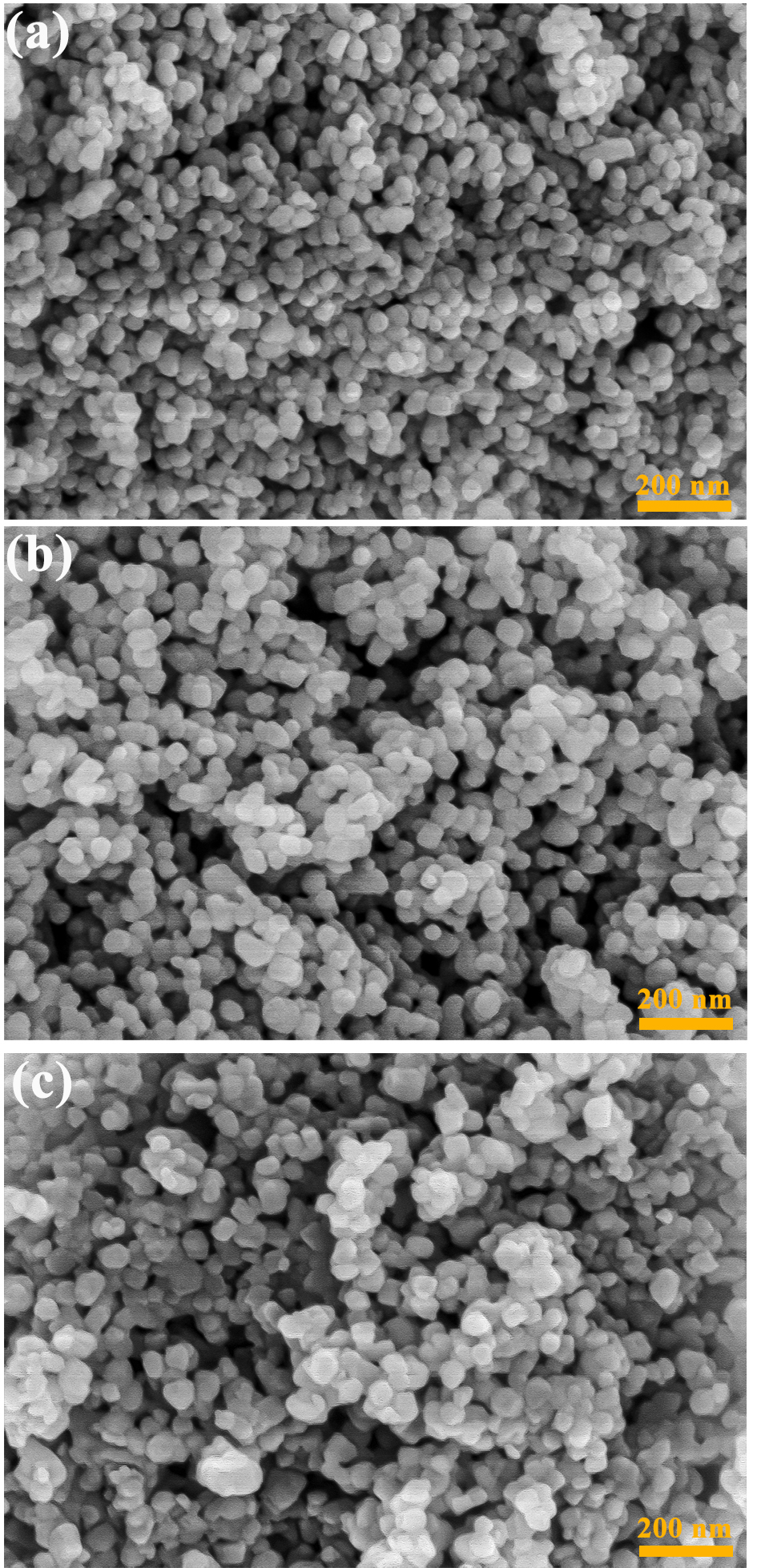


**Figure S3.** SEM images of (a) N_2.9_L_0.1_VOPF, (b) N_2.85_L_0.15_VOPF, and (c) N_2.8_L_0.2_VOPF.





**Figure S4.** Size distribution of (a) N_3_VOPF, (b) N_2.9_L_0.1_VOPF, (c) N_2.85_L_0.15_VOPF, and (d) N_2.8_L_0.2_VOPF.





**Figure S5.** Size distribution of N_2.85_L_0.15_VOPF@NC-1/2/3.


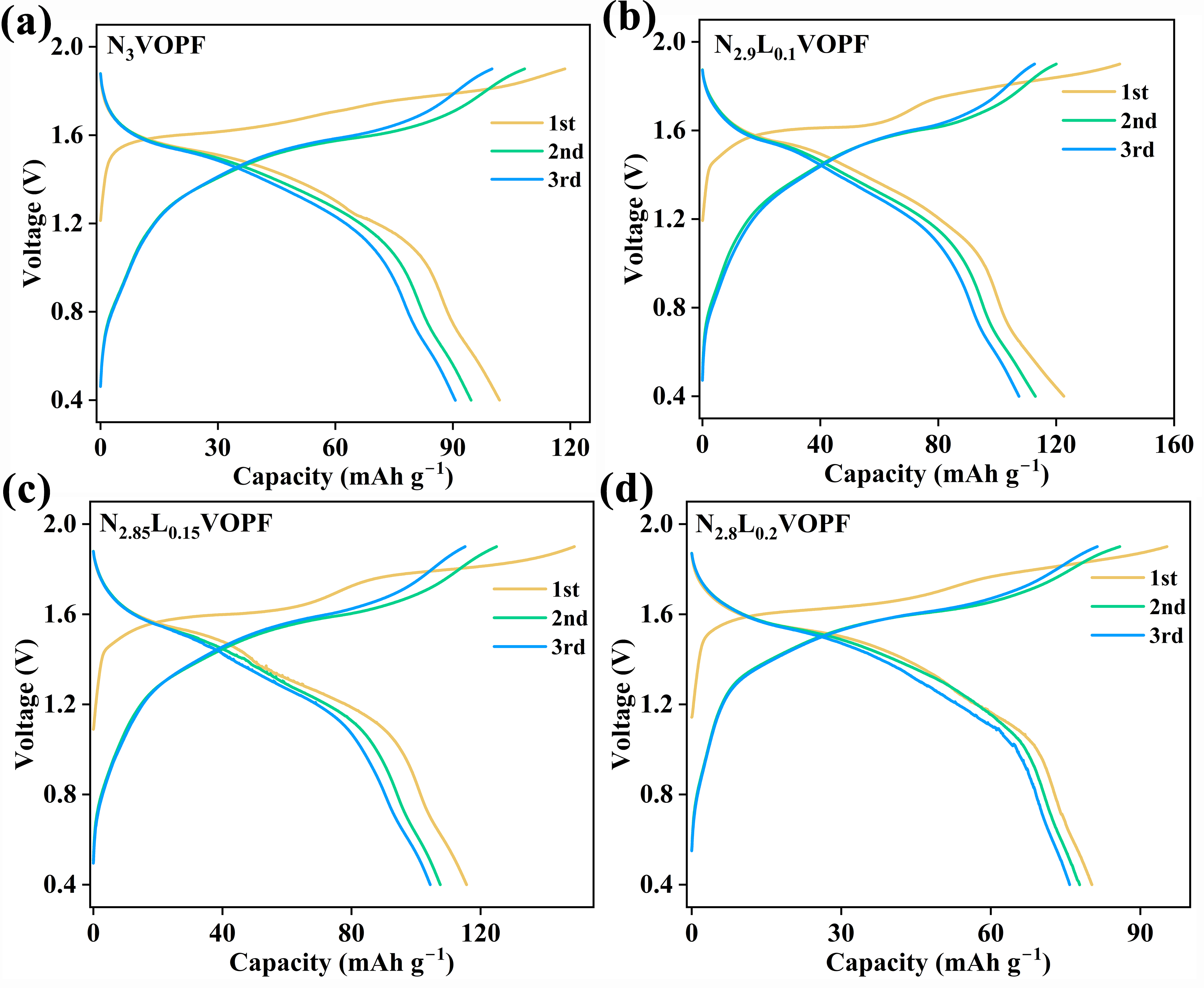


**Figure S6.** GCD profiles for the first three cycles at 0.1 A g^−1^. (a) N_3_VOPF, (b) N_2.9_L_0.1_VOPF, (c) N_2.85_L_0.15_VOPF, and (d) N_2.8_L_0.2_VOPF.


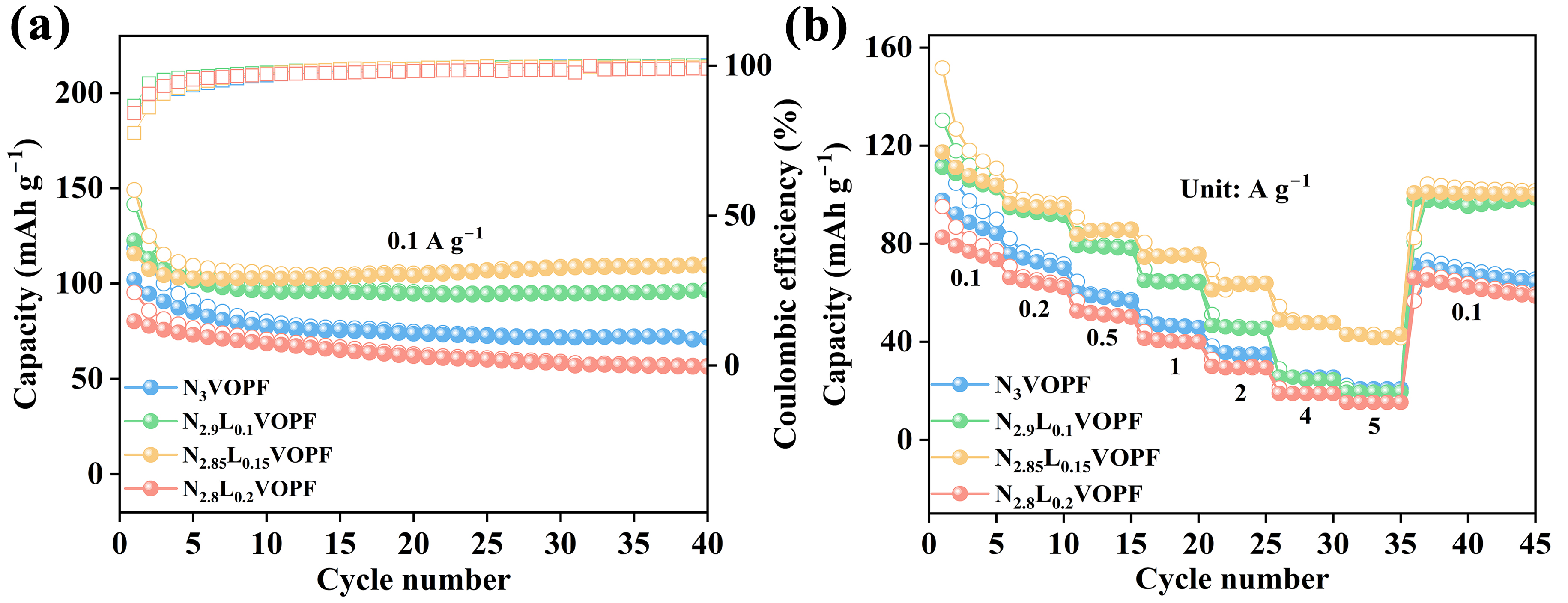


**Figure S7.** Electrochemical properties of N_3-x_L_x_VOPF. (a) Cycling performance at 0.1 A g^−1^. (b) Rate performance.

**
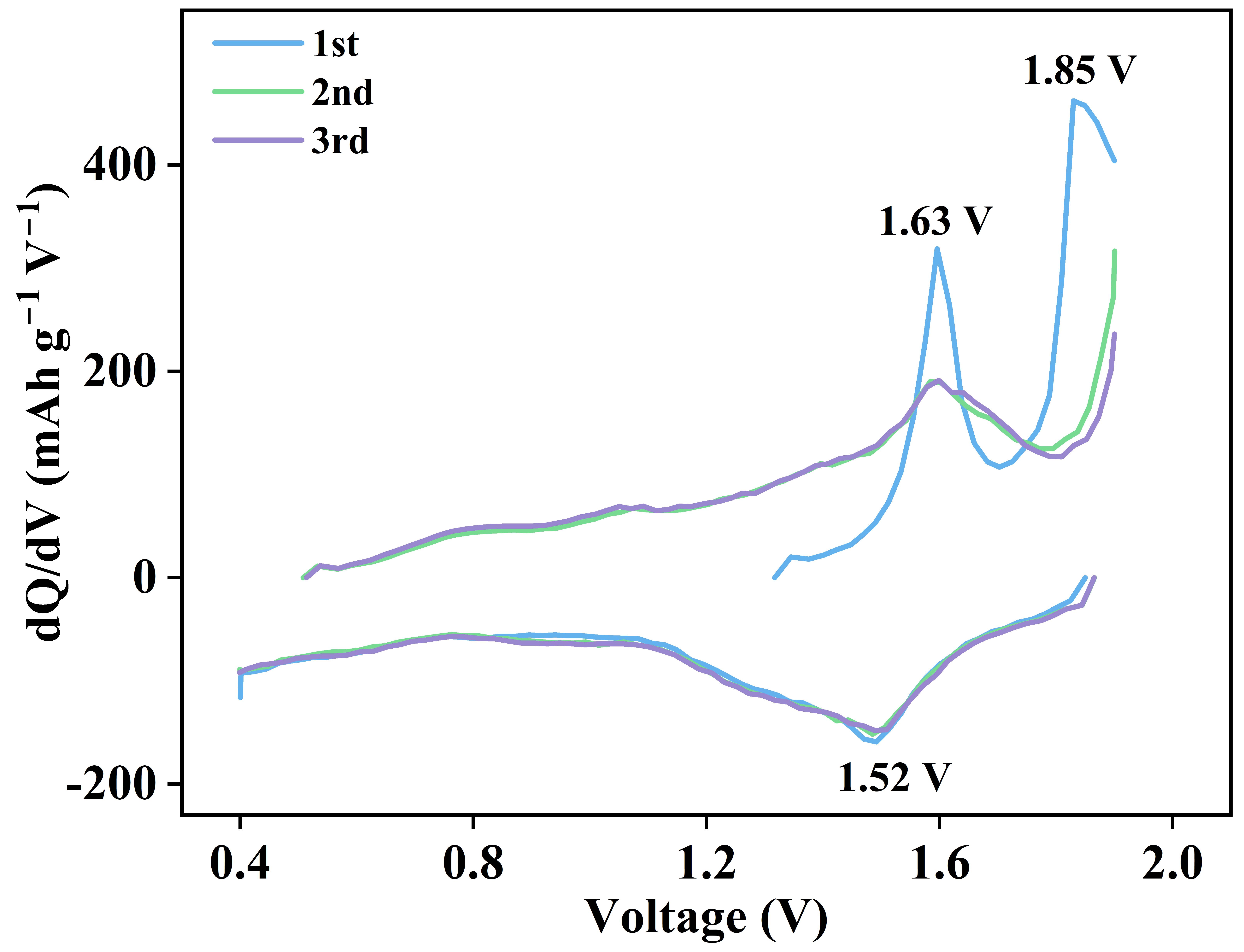
**

**Figure S8.** The dQ/dV curves of N_2.85_L_0.15_VOPF@NC-2.

**
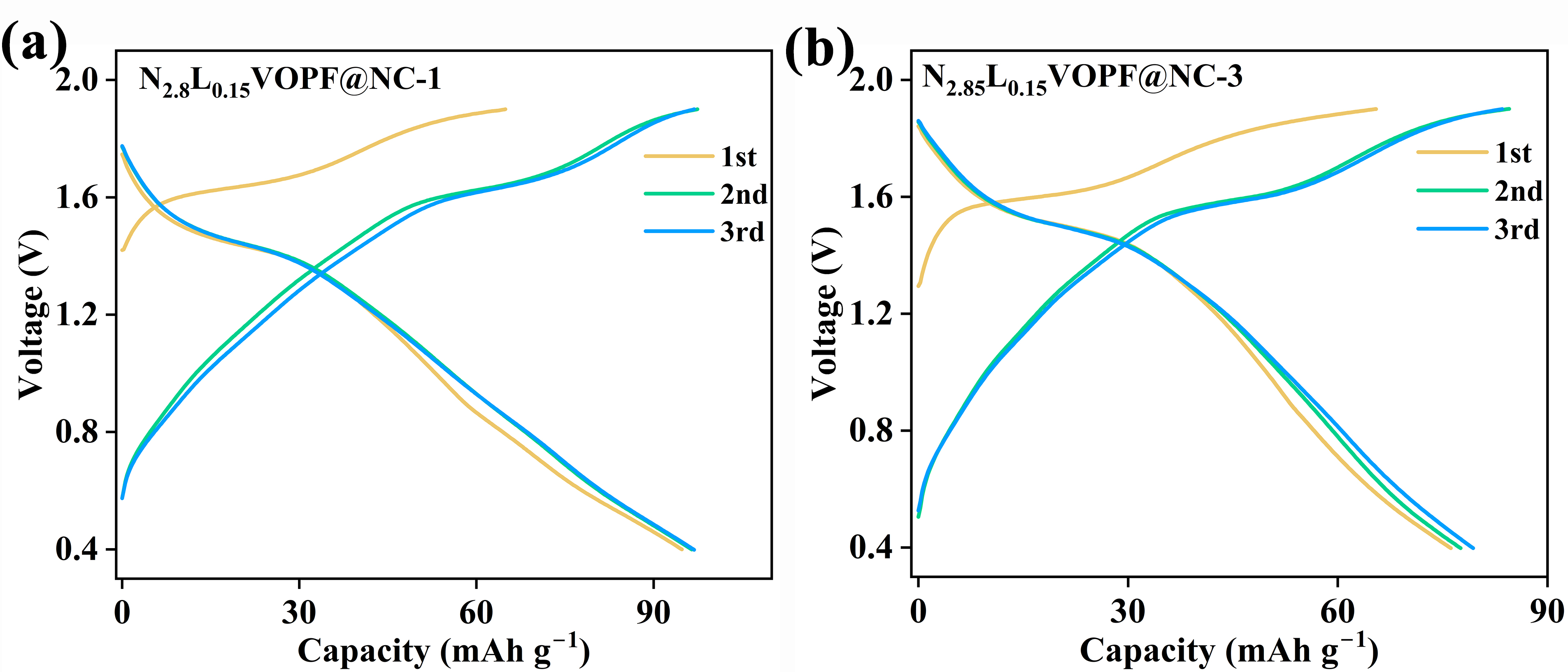
**

**Figure S9.** GCD profiles for the first three cycles at 0.5 A g^−1^. (a) N_2.85_L_0.15_VOPF@NC-1. (b) N_2.85_L_0.15_VOPF@NC-3.

**
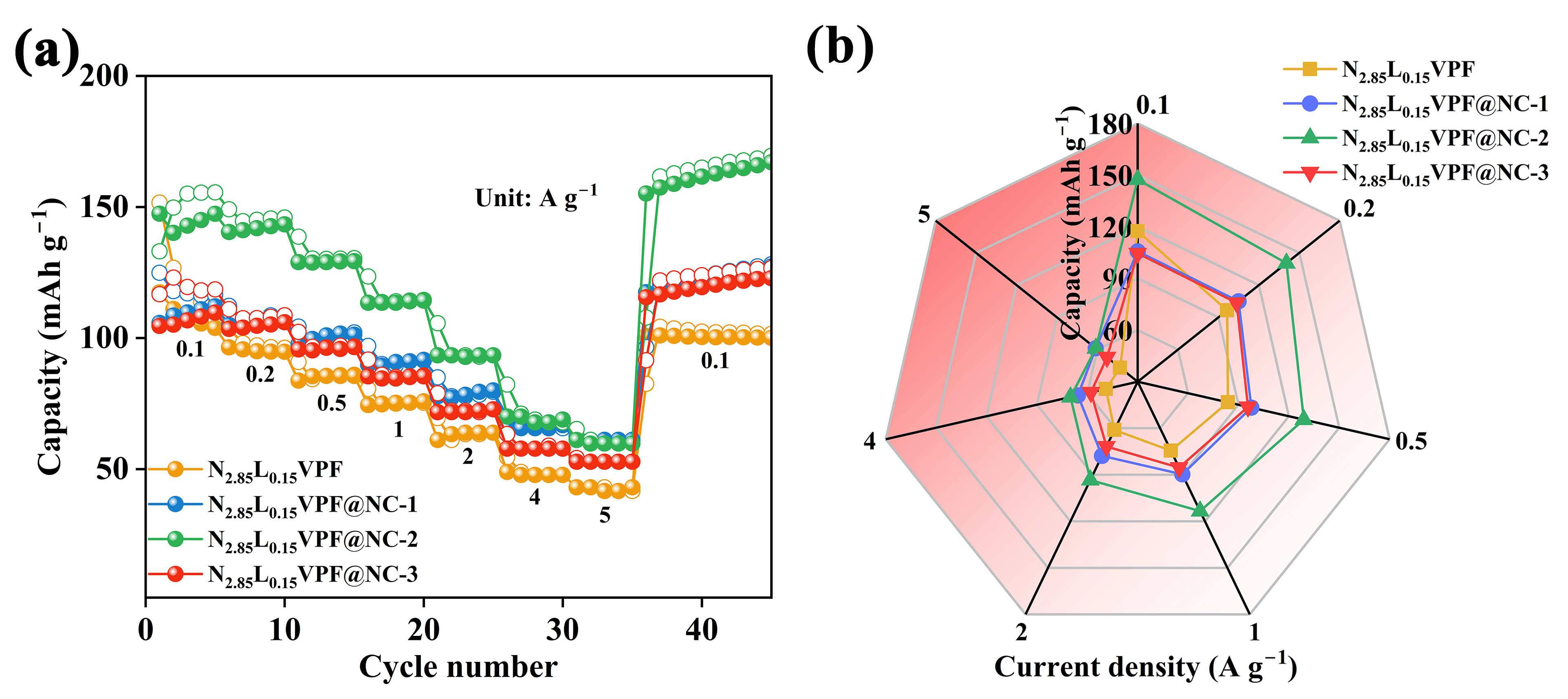
**

**Figure S10.** Rate performance. (a) Point plots. (b) Radargram.

**
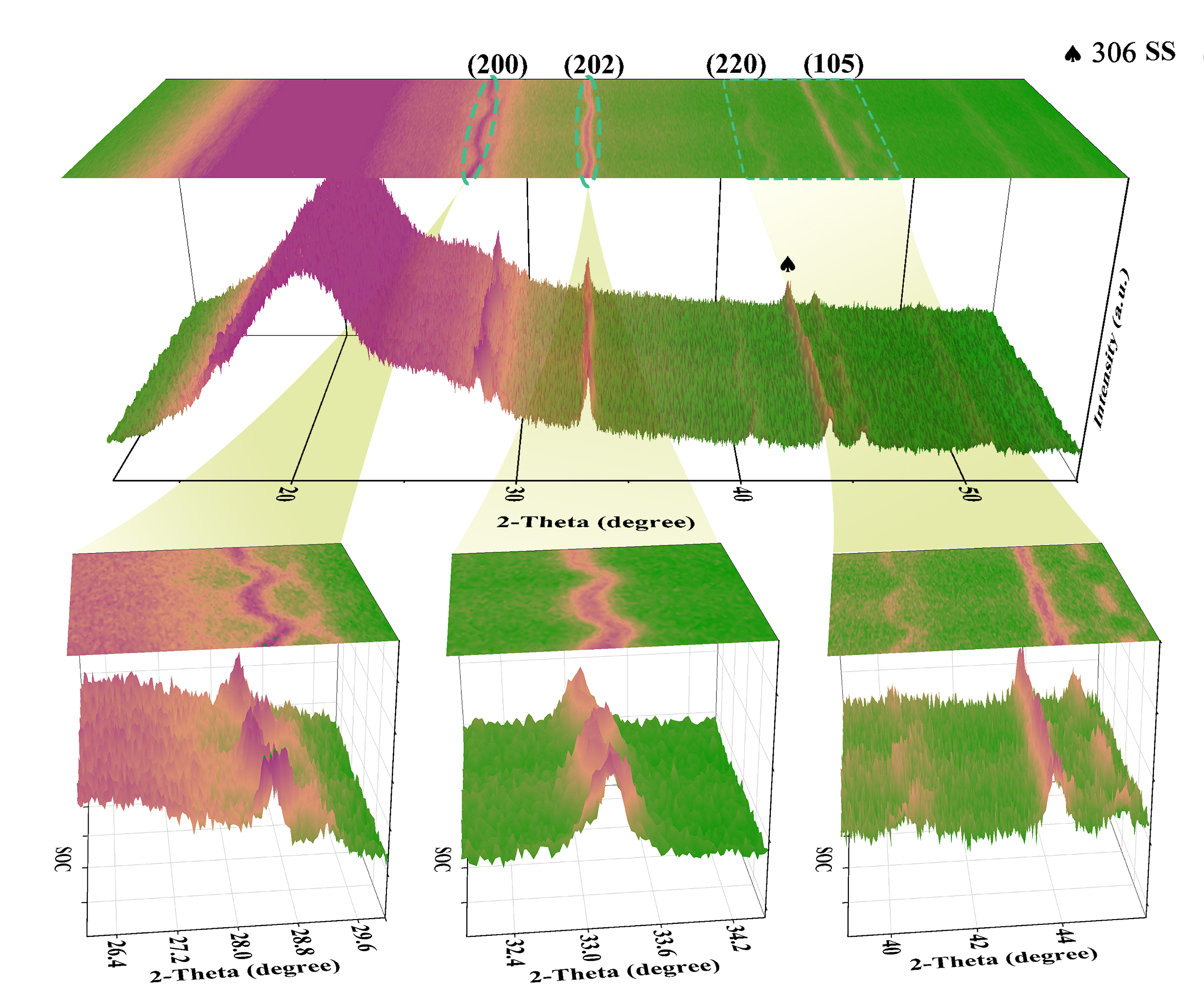
**

**Figure S11**. *In situ* XRD patterns.


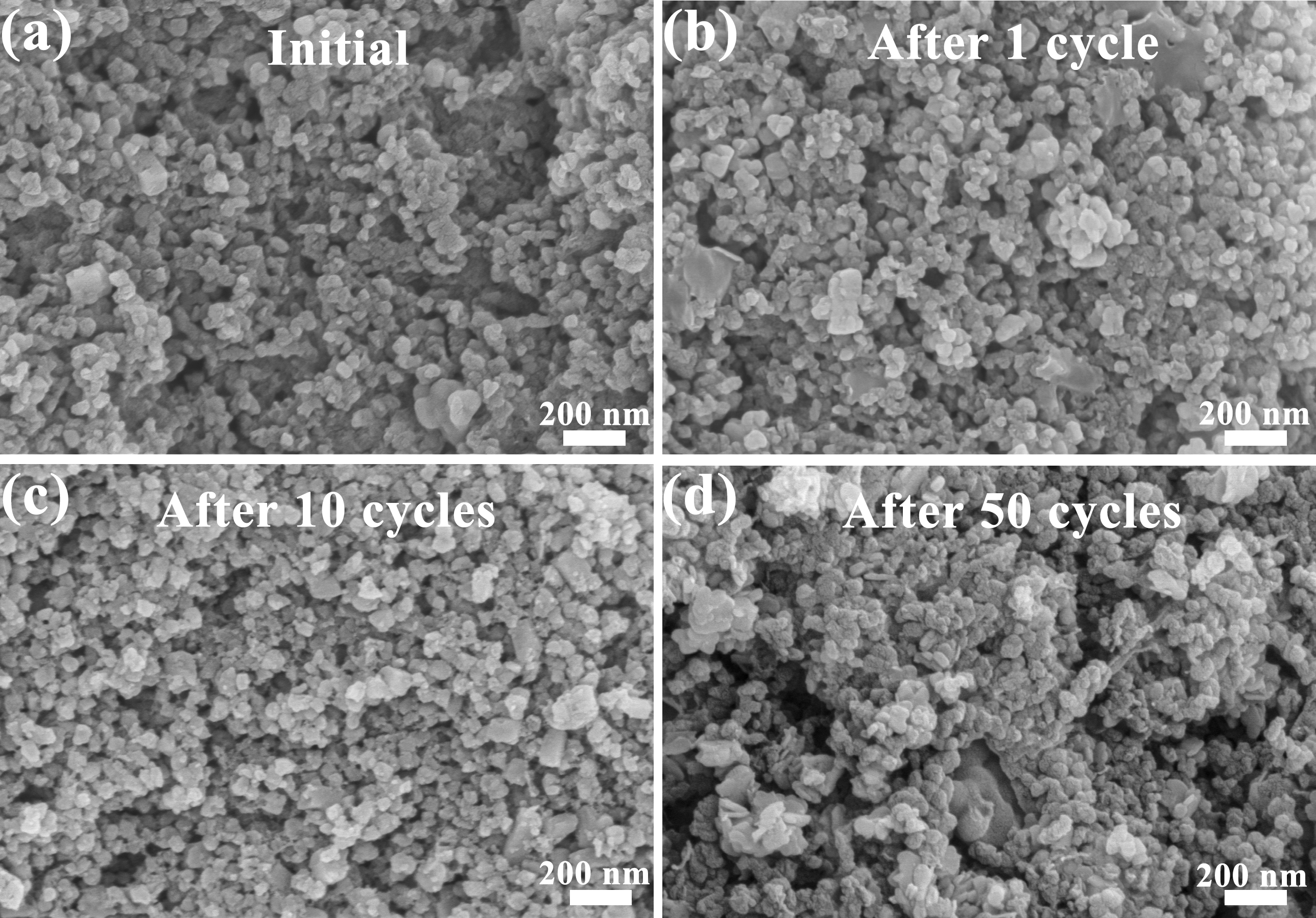


**Figure S12.** SEM images of N_2.85_L_0.15_VOPF@NC-2 electrodes cycled at 0.5 A g^−1^ for (a) initial, (b) 1^st^ cycle, (c) 10^th^ cycle, and (d) 50^th^ cycle states.


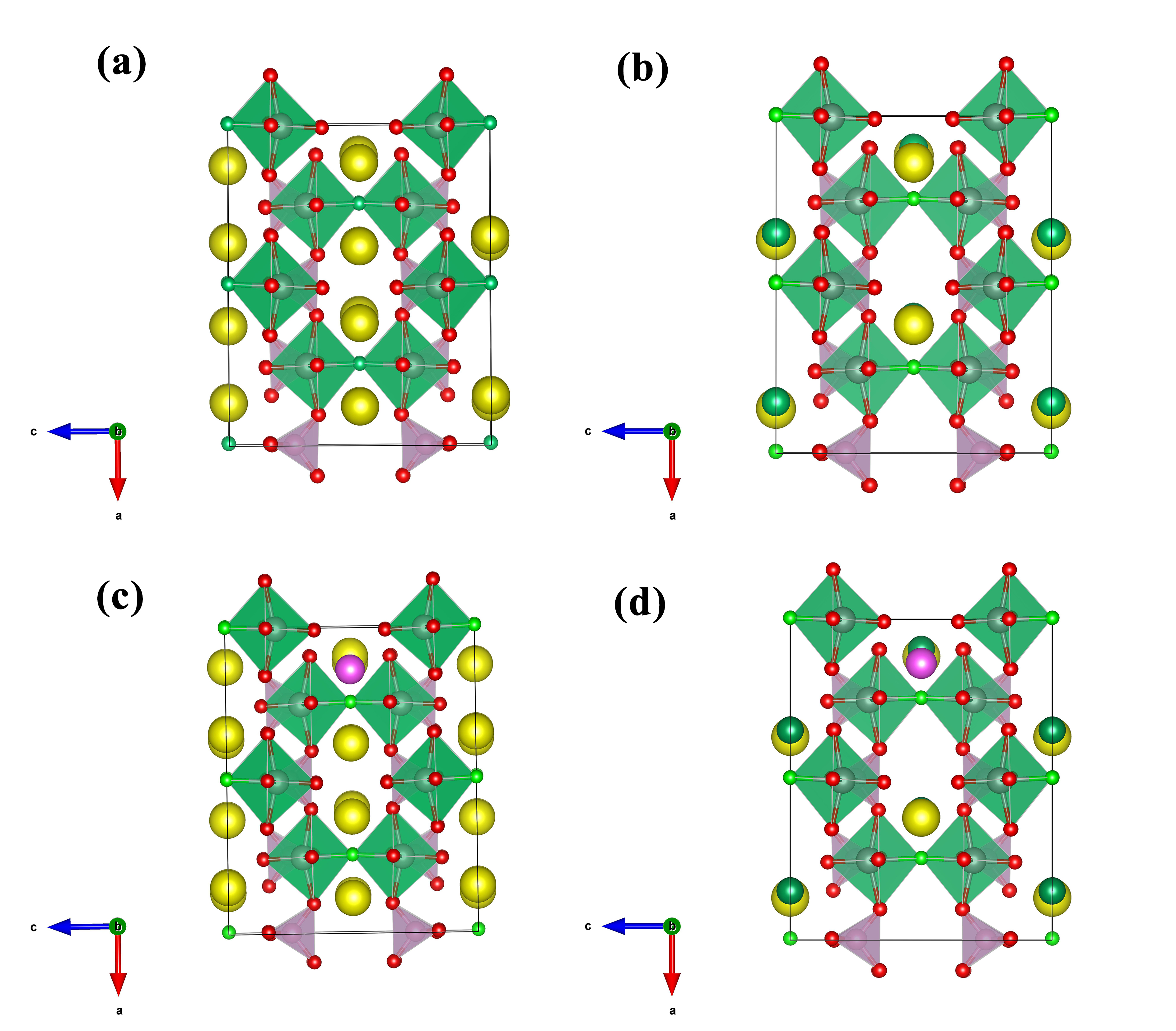


**Figure S13.** Optimized structure of pristine (a) N_3_VOPF and (c) N_2.85_L_0.15_VOPF. Optimized structure after inserting Zn^2+^ for (b) N_3_VOPF and (d) N_2.85_L_0.15_VOPF.


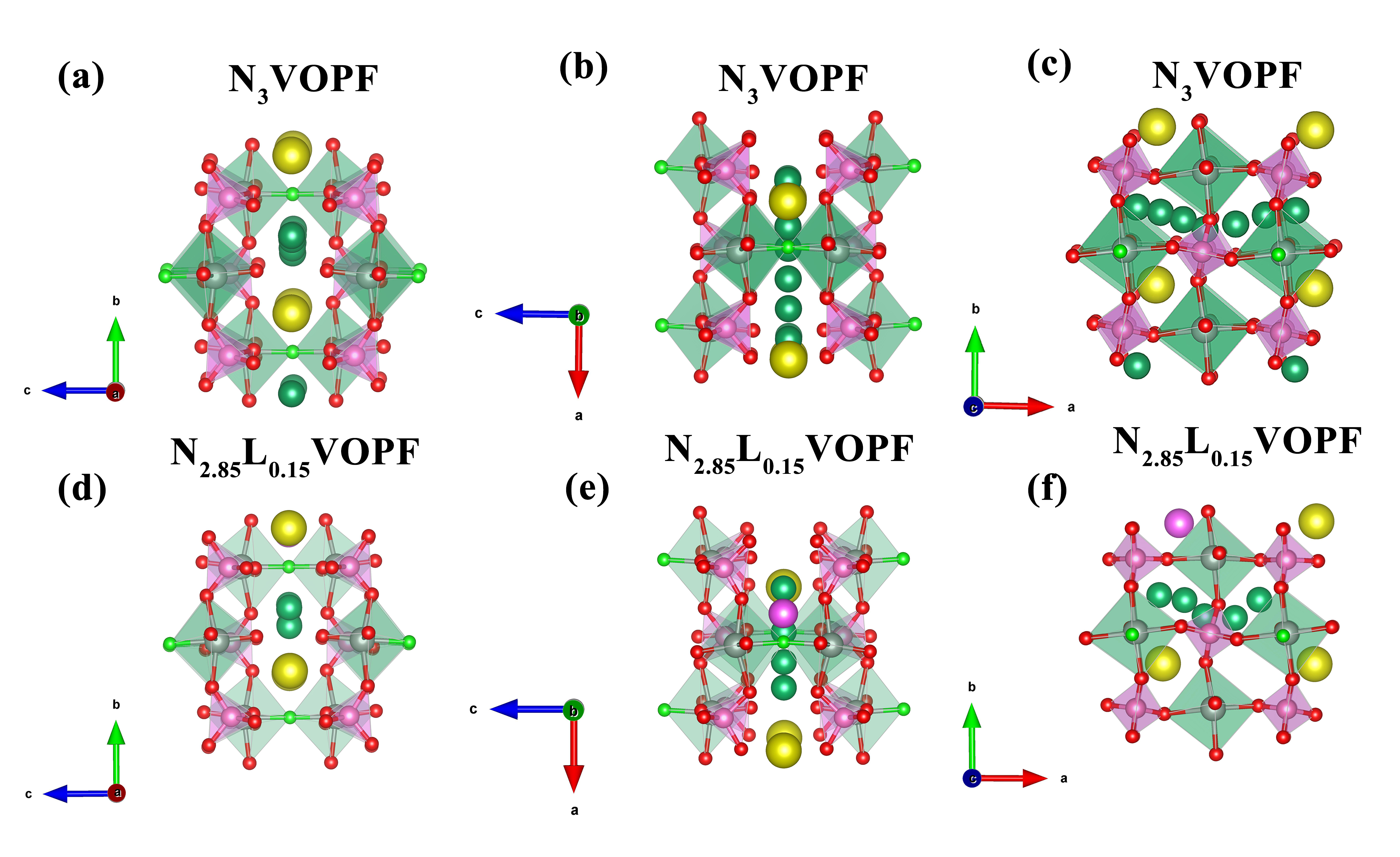


**Figure S14.** Zn^2+^ diffusion paths at different directions. (a–c) N_3_VOPF and (d–f) N_2.85_L_0.15_VOPF.

**
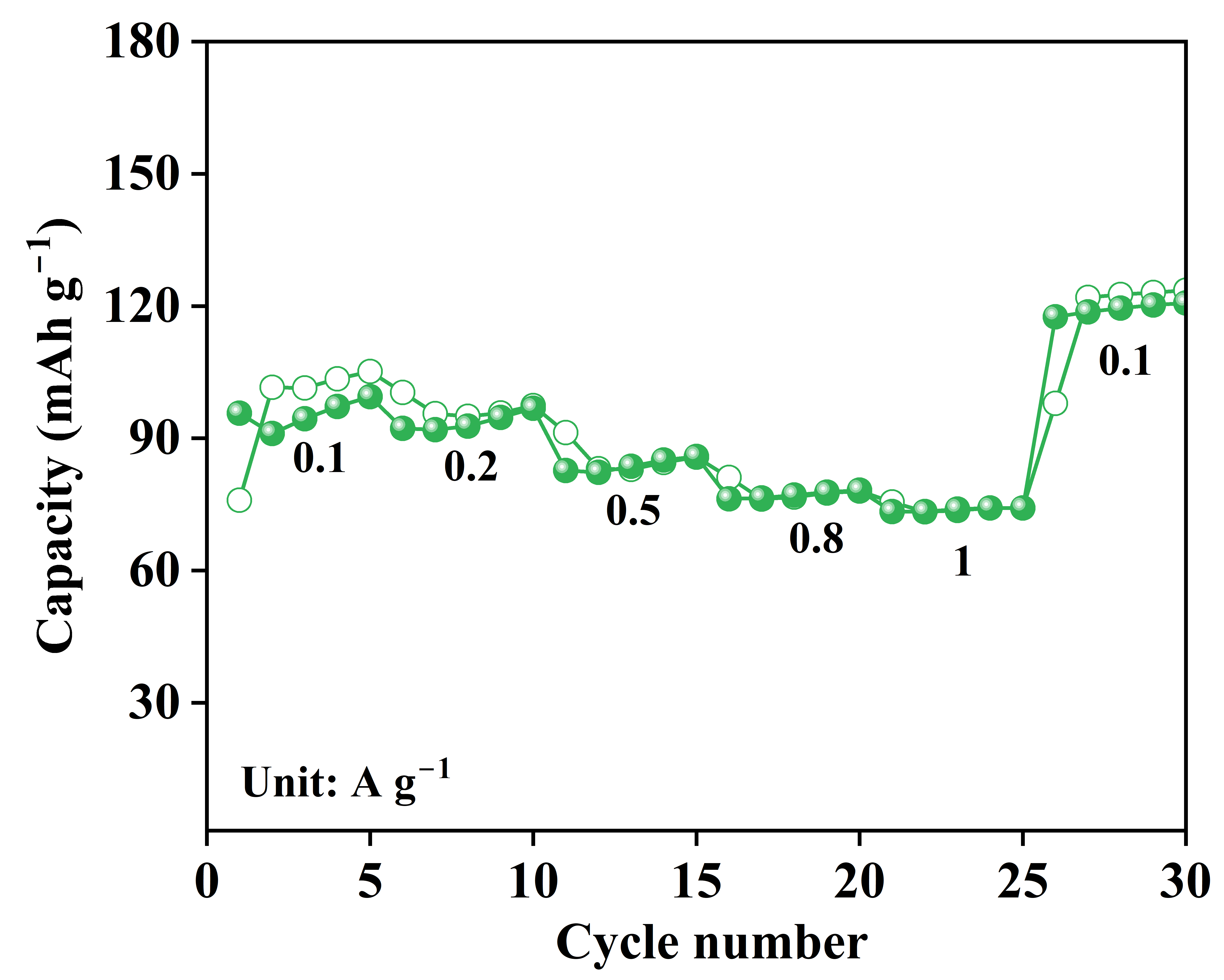
**

**Figure S15.** Rate performance of the soft package battery.


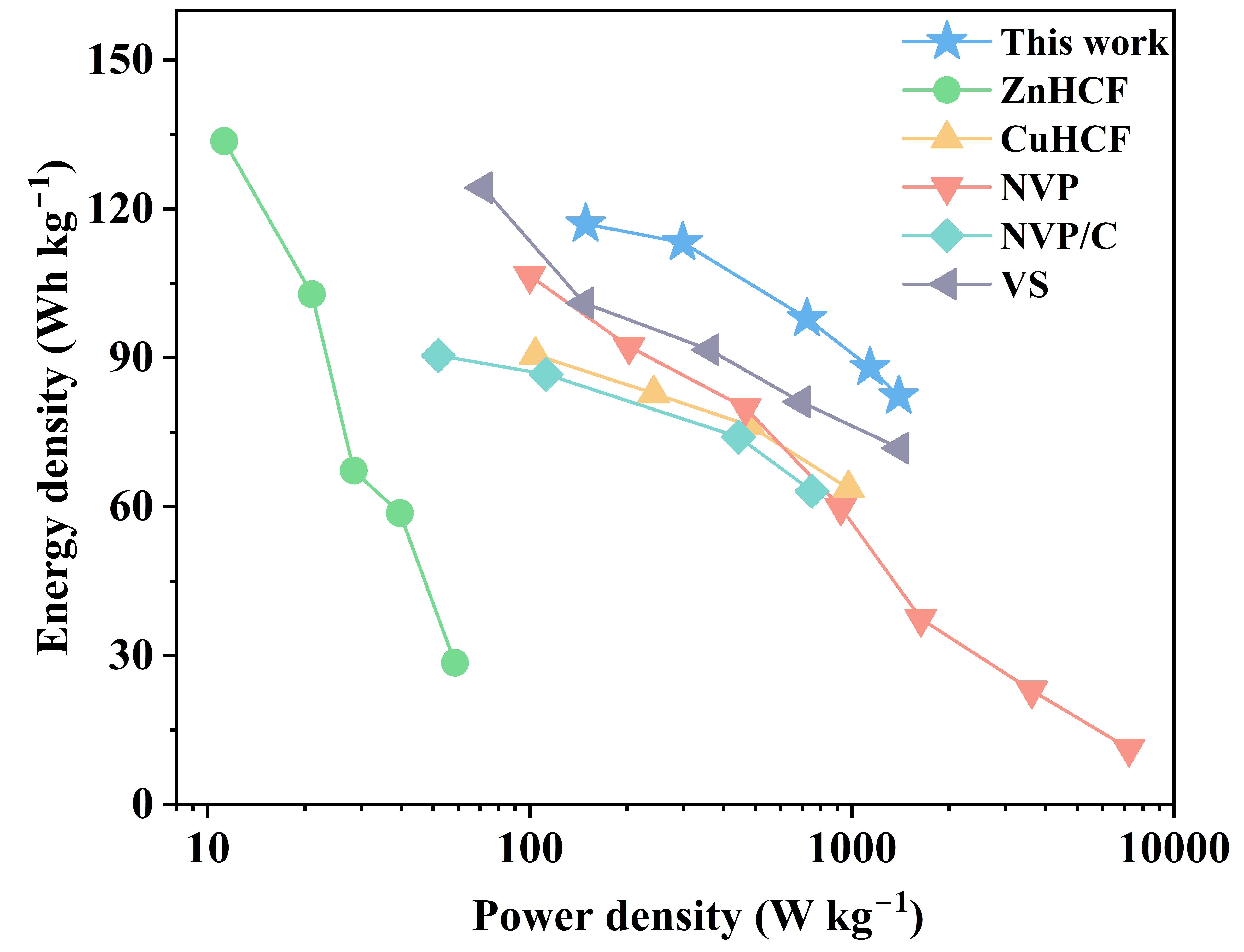


**Figure S16.** Ragone plots of N_2.85_L_0.15_VOPF@NC-2 soft package batteries in comparison with other reported cathode materials.^[5]^

**Table S1.** Refined atomic occupancies in N_3_VOPF.

| **Space group: *I4/mmm***. **a=b=6.39156 Å, c=10.63247 Å.**  **V=434.358 Å^3^** | | | | | |
| --- | --- | --- | --- | --- | --- |
| Name | x | y | z | Occ | Mult |
| O2 | 0.00000 | 0.00000 | 0.35655 | 0.125 | 4 |
| Na2 | -0.35358 | -0.24658 | 0.00000 | 0.095 | 16 |
| O1 | 0.31274 | 0.00000 | 0.16320 | 0.500 | 16 |
| P1 | 0.50000 | 0.00000 | 0.25000 | 0.125 | 4 |
| V1 | 0.00000 | 0.00000 | 0.19837 | 0.125 | 4 |
| Na1 | -0.25151 | -0.25151 | 0.00000 | 0.095 | 8 |
| F1 | 0.00000 | 0.00000 | 0.00000 | 0.062 | 2 |

**Table S2.** Refined atomic occupancies in N_2.9_L_0.1_VOPF.

| **Space group: *I4/mmm***. **a=b=6.38756 Å, c=10.63329 Å.**  **V=** **433.849 Å^3^** | | | | | |
| --- | --- | --- | --- | --- | --- |
| Name | x | y | z | Occ | Mult |
| O2 | 0.00000 | 0.00000 | 0.37278 | 0.125 | 4 |
| Na2 | -0.33547 | -0.23901 | 0.00000 | 0.095 | 16 |
| O1 | 0.31488 | 0.00000 | 0.16147 | 0.500 | 16 |
| P1 | 0.50000 | 0.00000 | 0.25000 | 0.125 | 4 |
| V1 | 0.00000 | 0.00000 | 0.19922 | 0.125 | 4 |
| Na1 | -0.26616 | -0.26616 | 0.00000 | 0.089 | 8 |
| F1 | 0.00000 | 0.00000 | 0.00000 | 0.063 | 2 |
| Li1 | -0.26616 | -0.26616 | 0.00000 | 0.006 | 8 |

**Table S3.** Refined atomic occupancies in N_2.85_L_0.15_VOPF.

| **Space group: *I4/mmm***. **a=b=** **6.37964 Å, c=** **10.63236 Å.**  **V=** **432.735 Å^3^** | | | | | |
| --- | --- | --- | --- | --- | --- |
| Name | x | y | z | Occ | Mult |
| O2 | 0.00000 | 0.00000 | 0.36266 | 0.124 | 4 |
| Na2 | -0.34609 | -0.23726 | 0.00000 | 0.095 | 16 |
| O1 | 0.31000 | 0.00000 | 0.16131 | 0.500 | 16 |
| P1 | 0.50000 | 0.00000 | 0.25000 | 0.125 | 4 |
| V1 | 0.00000 | 0.00000 | 0.19994 | 0.125 | 4 |
| Na1 | -0.26617 | -0.26617 | 0.00000 | 0.086 | 8 |
| F1 | 0.00000 | 0.00000 | 0.00000 | 0.062 | 2 |
| Li1 | -0.26617 | -0.26617 | 0.00000 | 0.009 | 8 |

**Table S4.** Refined atomic occupancies in N_2.8_L_0.2_VOPF.

| **Space group: *I4/mmm***. **a=b=** **6.37683 Å, c=** **10.63467 Å.**  **V=** **432.448 Å^3^** | | | | | |
| --- | --- | --- | --- | --- | --- |
| Name | x | y | z | Occ | Mult |
| O2 | 0.00000 | 0.00000 | 0.35586 | 0.125 | 4 |
| Na2 | -0.32701 | -0.24979 | 0.00000 | 0.095 | 16 |
| O1 | 0.31233 | 0.00000 | 0.16142 | 0.500 | 16 |
| P1 | 0.50000 | 0.00000 | 0.25000 | 0.125 | 4 |
| V1 | 0.00000 | 0.00000 | 0.19900 | 0.125 | 4 |
| Na1 | -0.25058 | -0.25058 | 0.00000 | 0.082 | 8 |
| F1 | 0.00000 | 0.00000 | 0.00000 | 0.062 | 2 |
| Li1 | -0.25058 | -0.25058 | 0.00000 | 0.013 | 8 |

**Table S5.** ICP-MS results of N_2.85_L_0.15_VOPF.

| Sample | V (ug L^−1^) | Li (ug L^−1^) |
| --- | --- | --- |
| N_2.85_L_0.15_VOPF | 5.014 | 0.071 |

**Table S6.** Electronic conductivity of N_2.85_L_0.15_VOPF and N_2.85_L_0.15_VOPF@NC-2.

| Sample | N_2.85_L_0.15_VOPF | N_2.85_L_0.15_VOPF@NC-2 |
| --- | --- | --- |
| Average electronic conductivity (S cm^−1^) | 2.26×10^−5^ | 3.75×10^−5^ |

References:

[1] a) G. Kresse, J. Hafner, *Phys. Rev. B* **1993**, 47, 558; b) G. Kresse, J. Furthmüller, *Phys. Rev. B* **1996**, 54, 11169.

[2] a) G. Kresse, D. Joubert, *Phys. Rev. B* **1999**, 59, 1758; b) P. E. Blochl, *Phys. Rev. B* **1994**, 50, 17953.

[3] J. P. Perdew, K. Burke, M. Ernzerhof, *Phys. Rev. Lett.* **1996**, 77, 3865.

[4] a) G. Henkelman, B. P. Uberuaga, H. Jónsson, *J. Chem. Phys.* **2000**, 113; b) G. Henkelman, H. Jónsson, *J. Chem. Phys.* **2000**, 113.

[5] a) N. Zhang, X. Y. Chen, M. Yu, Z. Q. Niu, F. Y. Cheng, J. Chen, *Chem. Soc. Rev.* **2020**, 49, 4203; b) L. Zhang, L. Chen, X. Zhou, Z. Liu, *Adv. Energy Mater.* **2015**, 5, 1400930; c) G. l. Li, Z. Yang, Y. Jiang, C. h. Jin, W. Huang, X. l. Ding, Y. h. Huang, *Nano Energy* **2016**, 25, 211; d) R. Trocoli, F. La Mantia, *ChemSusChem* **2015**, 8, 481; e) Z. Y. Wu, F. Ye, Q. Liu, R. Lv, J. Pang, Y. Liu, L. Jiang, Z. L. Tang, L. F. Hu, *Adv. Energy Mater.* **2022**, 12, 2200654.
